# AdipoR1–AMPK axis suppresses breast cancer across molecular subtypes via multimodal cell death pathways, including ferroptosis and apoptosis

**DOI:** 10.1038/s41419-026-08583-7

**Published:** 2026-03-26

**Authors:** Shinya Sato, Takashi Yamanaka, Yukako Komori, Mutsumi Ishida, Yoshiyasu Nakamura, Toshinari Yamashita, Yohei Miyagi

**Affiliations:** 1 Morphological Analysis Laboratory, Kanagawa Cancer Center Research Institute, Yokohama, Kanagawa Japan; 2 Molecular Pathology and Genetics Division, Kanagawa Cancer Center Research Institute, Yokohama, Kanagawa Japan; 3 Department of Pathology, Kanagawa Cancer Center, Yokohama, Kanagawa Japan; 4 Department of Breast Surgery and Oncology, Kanagawa Cancer Center, Yokohama, Kanagawa Japan

**Keywords:** Breast cancer, Translational research

## Abstract

Adipokines secreted by adipocytes have emerged as critical modulators of cancer progression, particularly in obesity-associated malignancies. However, their therapeutic relevance and the tumor types responsive to adipokine pathways remain unclear. To identify adipokine-driven cancers and assess the therapeutic potential of adipokine signaling, we conducted a pan-cancer transcriptome analysis of the expression of various adipokine receptors in 31 tumor types. AdipoR1 was most frequently amplified and overexpressed in breast cancer across molecular subtypes. In the functional analysis, AdipoR1 stimulation using the agonist AdipoRon activated AMPK signaling, suppressed proliferation and migration, and induced apoptosis in both hormone receptor (HR)-positive (MCF7, T47D) and triple-negative (MDA-MB-231, MDA-MB-468) breast cancer cells. Notably, RNA-Seq analysis revealed that AdipoR1 stimulation upregulated ferroptosis-related genes, DDIT3, HMOX1, and IRE1α, and downregulated proliferation-related genes, estrogen receptor and TROP2, in breast cancer cell lines. Immunoblotting confirmed these changes at the protein level. AdipoR1 activation enhanced the efficacy of chemotherapeutic agents. In vivo, AdipoRon significantly reduced tumor growth and induced necrotic cell death. AdipoR1 activation exerts multimodal antitumor effects by engaging cell death and hormone receptor signaling. These findings establish AdipoR1 as a valuable therapeutic target in breast cancer and support further development of adipokine receptor-targeting therapies.

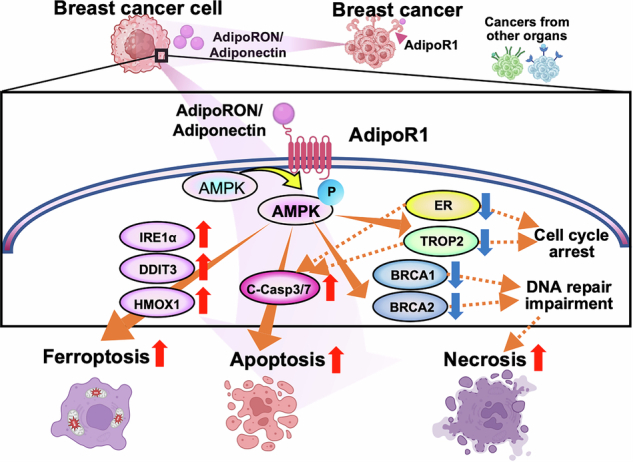

## Introduction

The tumor microenvironment (TME) comprises cancer cells surrounded by organ-specific resident and stromal cells such as immune cells, blood vessel cells, fibroblasts, neurons, myocytes, and adipocytes [1–3]. In recent years, the importance of stromal cells interacting with cancer cells in cancer progression has become increasingly evident [4–6]. The cancer-regulatory mechanisms of immune cells, blood vessel cells, and fibroblasts have been identified, and drugs targeting cancer–stromal interaction mechanisms have been applied in standard cancer therapy [7–10]. The mechanism of action of therapies targeting stromal cells, such as immune and blood vessel cells, differs from that of conventional therapies targeting cancer cells and exerts additive effects when combined with cancer-targeting therapies [11–14]. Therapies targeting stromal cells are essential for improving the efficacy of cancer treatment; however, the roles of certain stromal cells in the TME are still not fully understood, and further research is required to advance cancer treatment.

Adipocytes are stromal cells found extensively throughout the body, except in the brain, and they exist in the TME along with cancer cells at nearly all primary and metastatic cancer sites. Adipocytes not only interact directly with cancer cells, but they also secrete various factors that influence cancer progression [15]. Adipokines are secreted factors unique to adipocytes. More than 30 types of adipokines, including adiponectin and leptin, have been identified and functionally analyzed [16–18]. Certain adipose-derived secretory factors reportedly affect cancer growth; however, most have only been studied in preliminary in vitro studies using cell lines or in epidemiological studies [19–27]. Moreover, the detailed molecular mechanisms by which adipokines control cancer growth and progression remain understudied, and whether the genetic background and histological characteristics of cancer affect these mechanisms remains unknown. For the practical application of adipokine-targeted cancer therapy, cellular and animal model studies are required to elucidate and validate these mechanisms. Furthermore, to optimize treatment, it is necessary to identify the specific types of cancer in which adipokines are important for proliferation control, as well as their genetic and molecular subtypes, by analyzing patient samples.

This study aimed to identify specific cancer types in which adipokines are involved in growth control and to elucidate the underlying molecular mechanisms. Using The Cancer Genome Atlas (TCGA) data, we analyzed the gene expression of approximately 30 adipokines and adipokine receptors in more than 8000 patients with cancers originating from various organs. We found that adiponectin receptor 1 (AdipoR1) was highly expressed in breast cancer across genetic subtypes. Next, based on database analysis results and our previous research on adiponectin-mediated cancer control [25, 26], we analyzed the effect of adiponectin receptor stimulation on cancer cell control using multiple breast cancer cell lines of different genetic and molecular subtypes. Furthermore, we examined the cancer control effects of adiponectin receptor stimulation in combination with existing therapeutic drugs. Finally, we examined the effect of adiponectin receptor stimulation on breast cancer proliferation in vivo. This study reveals that adiponectin receptor stimulation is a novel therapeutic modality that transcends genetic and molecular breast cancer subtypes and demonstrates that controlling adipocytes and their secretory factors is important for breast cancer treatment.

## Results

### Pan-cancer analysis to identify cancer types expressing adipokine receptors

**Fig. 1:**
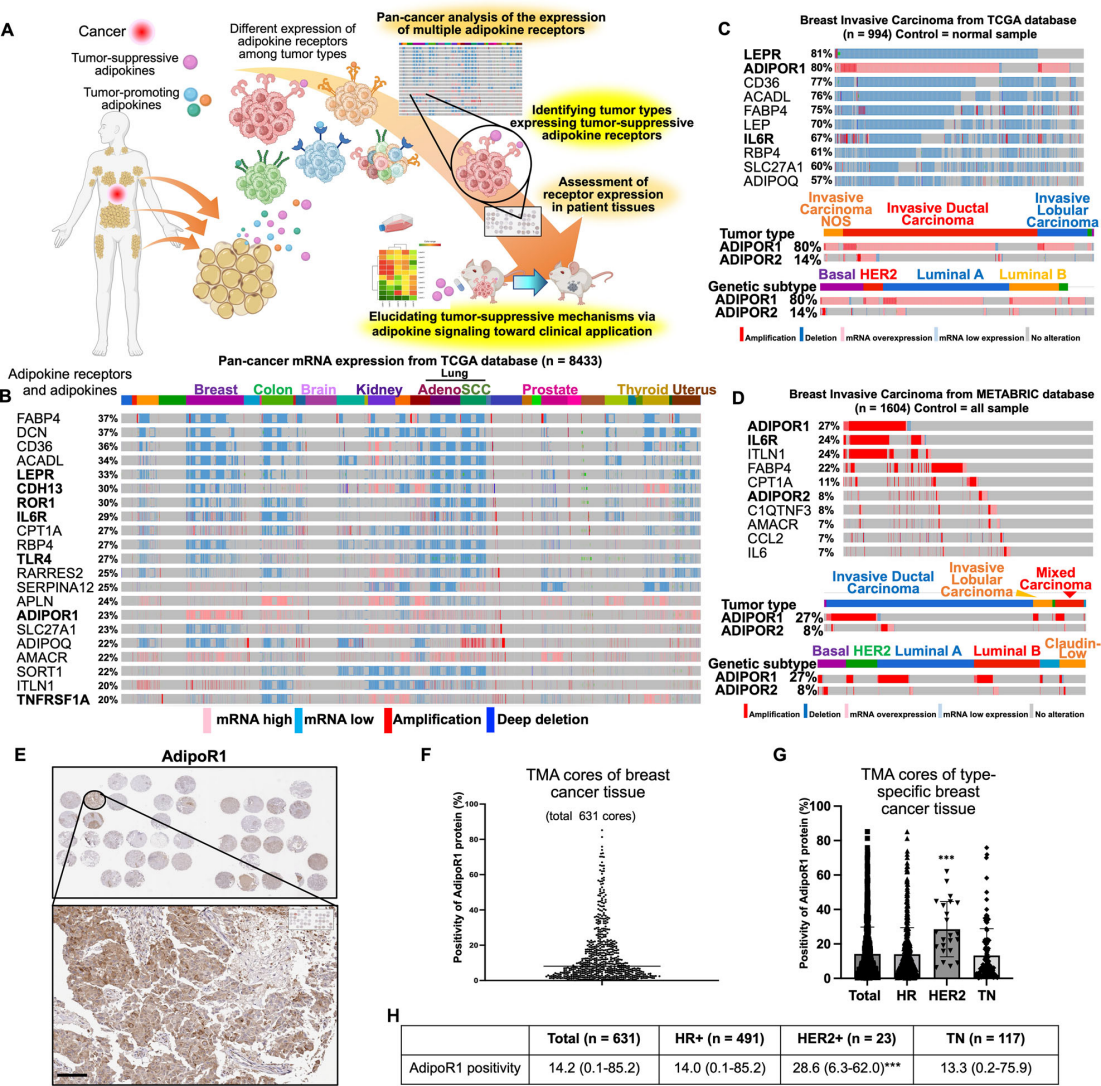
Pan-cancer adipokine expression analysis using large public databases reveals specific expression of adipokine receptors in breast cancer. **A** Overview of the study concept and design. **B** Global adipokine signaling-related gene expression differences in whole tumor tissues versus normal tissues in the TCGA database (*n* = 8433) in cBioPortal. From left to right: *ADIPOR1* mRNA expression, DNA amplification, and DNA deletion in acute myeloid leukemia, adrenocortical carcinoma, bladder urothelial carcinoma, brain lower grade glioma, breast invasive carcinoma, cervical squamous cell carcinoma, cholangiocarcinoma, colorectal adenocarcinoma, diffuse large B-cell lymphoma, esophageal adenocarcinoma, glioblastoma multiforme, head and neck squamous cell carcinoma, kidney chromophobe carcinoma, kidney renal clear cell carcinoma, kidney renal papillary cell carcinoma, liver hepatocellular carcinoma, lung adenocarcinoma, lung squamous cell carcinoma, mesothelioma, ovarian serous cystadenocarcinoma, pancreatic adenocarcinoma, pheochromocytoma and paraganglioma, prostate adenocarcinoma, sarcoma, skin cutaneous melanoma, stomach adenocarcinoma, testicular germ cell tumors, thymoma, thyroid carcinoma, uterine carcinosarcoma, uterine corpus endometrial carcinoma, and uveal melanoma were identified. The top 21 differentially expressed, amplified, and/or deleted genes among 37 adipokine signaling-related genes are shown. **C** Adipokine signaling-related gene expression differences in breast cancer tissues versus normal tissues in the TCGA database (*n* = 994) in cBioPortal. The top 10 differentially expressed adipokine signal-related genes in breast cancer tissue versus normal breast tissue are shown. The lower panel shows *ADIPOR1* and *ADIPOR2* expression rates and mutation by histopathological and genetic subtypes. **D** Adipokine signal-related gene expression differences in breast cancer tissues versus diploid samples in the METABRIC database (*n* = 1604) in cBioPortal. The top 10 differentially expressed adipokine signal-related genes in breast cancer tissue versus normal breast tissue are shown. The lower panel shows the *ADIPOR1* and *ADIPOR2* expression rates and mutations by histopathological and genetic subtypes. **E** Immunohistochemical staining of AdipoR1 in breast cancer tissue using tissue microarrays (TMAs). Representative images of anti-AdipoR1-stained (brown) invasive fronts are shown. **F** Percentage of AdipoR1-positive cells per TMA core. **G**, **H** Average percentage of AdipoR1-positive cells in TMA cores of various breast cancer subtypes. ***P < 0.001; one-way ANOVA followed by Tukey’s multiple comparison tests.

To identify cancer types in which adipokine receptors may serve as therapeutic targets and to elucidate the mechanisms of cancer suppression by adipokine signaling, we conducted experiments using public datasets, clinical tissues, cell lines, and animal models (Fig. 1A). First, we aimed to identify cancer types in which adipocyte-secreted factors, including adipokines and lipid metabolism-activating factors, are deeply involved in cancer progression. To this end, we analyzed the mRNA expression, DNA amplification, and deletion of 37 genes involved in adipokine recognition, adipokine signaling activation, and β-oxidation (Table 1) in 8433 pan-cancer cases across 31 tumor types, using data from the TCGA database in cBioPortal. We found that adipokine receptor expression fluctuated in breast, colon, liver, and lung cancers, whereas little fluctuation was observed in brain tumors (Fig. 1B). This finding corroborates that central nervous system tumors do not depend on adipokine signaling because the central nervous system is poor in adipocytes [28–30], and this validates the present analysis. We then aimed to identify cancers with significantly altered adipokine receptor expression. Among all cancers, the expression of many adipokine receptors and their ligands was altered in breast, colon, kidney, liver, lung, thyroid, and uterine cancers. In these cancers, most adipokine receptor and ligand genes with altered expression, such as *ACADL*, *DCN*, *FABP4*, and *LEPR*, were downregulated, whereas *ADIPOR1* and *APLN* were upregulated. We decided to focus on breast cancer, in which multiple adipokine receptors showed significantly altered expression, including widespread upregulation of *ADIPOR1*.

**Table 1:** List of adipokines and adipokine receptors analyzed in the present study.

Gene symbol	Gene name	Functions
*ACADL*	Acyl-CoA dehydrogenase long chain	Enzyme involved in fatty acid metabolism
*ADIPOQ*	Adiponectin	Adipokine
*ADIPOR1*	Adiponectin receptor 1	Adipokine receptor
*ADIPOR2*	Adiponectin receptor 2	Adipokine receptor
*AMACR*	Alpha-methylacyl-CoA racemase	Beta-oxidation-related enzyme
*APLN*	Apelin	Adipokine
*APLNR*	Apelin receptor	Adipokine receptor
*C1QTNF3*	C1q and TNF-related protein 3	Adipokine
*CD36*	CD36	Overexpressed in adipose tissue
*CCL2*	C-C Motif chemokine ligand 2	Adipokine
*CCR2*	C-C Motif chemokine receptor 2	Adipokine receptor
*CCR4*	C-C Motif chemokine receptor 4	Adipokine receptor
*CDH13*	Cadherin 13	Associated with the adipokine level
*CMKLR2*	Chemerin chemokine-like receptor 2	Adipokine receptor
*CPT1A*	Carnitine palmitoyltransferase 1A	Enzyme related to fatty acid oxidation
*DCN*	Decorin	Adipokine
*FABP4*	Fatty acid binding protein 4	Fatty acid binding protein for transport
*GRN*	Granulin precursor	Adipokine
*IL6*	Interleukin 6	Cytokine
*IL6R*	Interleukin 6 receptor	Cytokine receptor
*ITLN1*	Intelectin 1	Adipokine
*LEP*	Leptin	Adipokine
*LEPR*	Leptin receptor	Adipokine receptor
*NAMPT*	Nicotinamide phosphoribosyltransferase	Adipokine (known as visfatin)
*PAQR3*	Progestin and AdipoQ receptor family member 3	Adipokine receptor
*RARRES2*	Retinoic acid receptor responder 2	Adipokine (known as chemerin)
*RBP4*	Retinol binding protein 4	Adipokine
*RETN*	Resistin	Adipokine
*ROR1*	Receptor tyrosine kinase-like orphan receptor 1	Adipokine receptor
*SERPINE1*	Serpin family E member 1	Adipokine
*SLC22A5*	Solute carrier family 22 member 5	Lipid metabolism
*SLC27A1*	Solute carrier family 27 member 1	Adipokine
*SORT1*	Sortilin 1	Lipid metabolism
*TLR4*	Toll-like receptor 4	Adipokine receptor
*TNF*	Tumor necrosis factor	Cytokine
*TNFRSF1A*	TNF receptor superfamily member 1A	Cytokine receptor
*TNFRSF1B*	TNF receptor superfamily member 1B	Cytokine receptor

### AdipoR1 mRNA and protein are widely expressed in breast cancer across molecular and histological subtypes

Using TCGA data from breast cancer patients alone, we comprehensively analyzed expression changes in adipokine receptors in breast cancer versus normal breast tissue. *ADIPOR1* was highly expressed in 78% of all breast cancers. Further analysis revealed that *ADIPOR1* mRNA was overexpressed in more than 50% of cases of all four major gene subtypes, including luminal A and B, HER2, and triple-negative (TN), and in more than 50% of cases of both invasive ductal and lobular carcinoma, the major histological breast cancer types (Fig. 1C). The reproducibility of these results was verified using another dataset from the METABRIC database, in which the average of all cancer samples is used as a control instead of normal tissues. *ADIPOR1* DNA amplification or mRNA overexpression was observed in 27% of breast cancers, and *ADIPOR1* was the most frequently amplified and overexpressed gene among the 37 genes analyzed (Fig. 1D). Further analysis using yet another dataset and two types of controls corroborated these findings (Supplementary Fig. S1A–D).

To comprehensively analyze the expression status of adiponectin receptor proteins in breast cancer, AdipoR1 immunostaining was performed on 632 breast cancer tissue cores from 210 anonymized patients. AdipoR1-positive cancer cells were observed in all groups, including the hormone receptor (HR) (estrogen receptor [ER] and progesterone receptor [PgR])-positive, human epidermal growth factor receptor 2 (HER2)-positive, and HR- and HER2-negative (TN) groups (Fig. 1E, F). The percentage of stained cancer cells was significantly higher in the HER2-positive group than in the other groups (Fig. 1G, H). Taken together, these results indicate that AdipoR1 is widely expressed in breast cancer tissues at both the mRNA and protein levels.

### AdipoR1 expression in human and mouse breast cancer cell lines

**Fig. 2:**
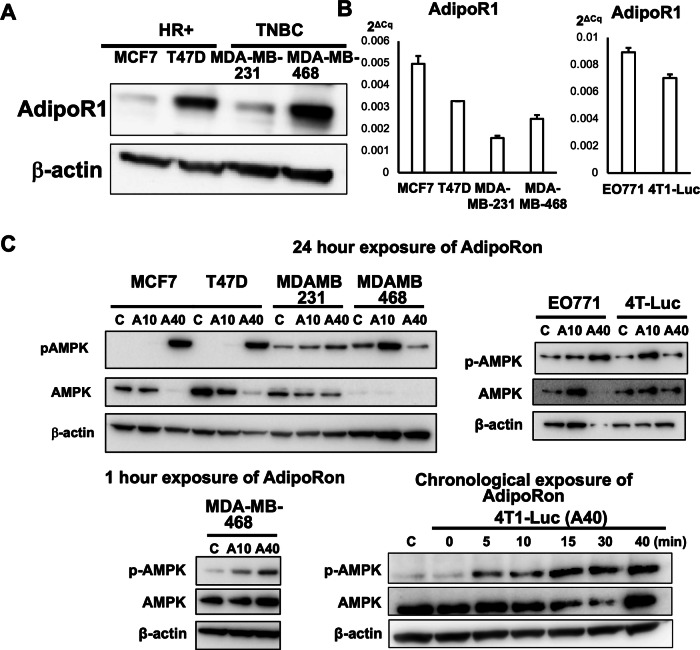
Breast cancer cell lines express AdipoR1, and AdipoRon activates AdipoR1 signaling. **A** AdipoR1 protein expression in MCF7, T47D, MDA-MB-231, and MDA-MB-468 human breast cancer cells. **B** *ADIPOR1* mRNA expression in MCF7, T47D, MDA-MB-231, and MDA-MB-468 human breast cancer cells and EO771 and 4T1-Luc mouse breast cancer cells. **C** Upper panel: pAMPK and AMPK protein expression in breast cancer cell lines 24 h after AdipoRon (40 µg/mL) exposure. Lower panel: time course of pAMPK and AMPK protein expression in breast cancer cell lines over 1 h after exposure to AdipoRon (40 µg/mL).

Although studies have shown that AdipoR1 stimulation regulates cancer cell proliferation [31–36], the detailed underlying mechanism in breast cancer remains unknown. Further, the significance of AdipoR1 signaling activation in breast cancers of different genetic and molecular subtypes remained unclear. Therefore, we investigated the effects of AdipoR1 stimulation on breast cancer cells using human breast cancer cell lines of various genetic and molecular subtypes, including HR-positive MCF7 and T47D cells and TN MDA-MB-231 and MDA-MB-468 cells. First, we confirmed the expression of AdipoR1 in each cell line. Data from the DepMap Portal (https://depmap.org/portal/ccle/) and the Human Protein Atlas (https://www.proteinatlas.org) confirmed *ADIPOR1* mRNA expression in all four cell lines (Supplementary Fig. S1E). In quantitative polymerase chain reaction (PCR) analysis, *ADIPOR1* mRNA expression trends in the four cell lines were similar to those in the public databases (Fig. 2A). Western blot analysis showed that AdipoR1 protein expression correlated with mRNA expression in all cell lines except MCF7 (Fig. 2B). Finally, we investigated *AdipoR1* expression in mouse breast cancer cell lines used in experiments, including TN 4T1-Luc mouse cells and HR-positive EO771 mouse cells. Both mouse cell lines expressed *AdipoR1* mRNA (Fig. 2A).

### The synthetic AdipoR1 agonist AdipoRon induces AMPK phosphorylation in breast cancer cell lines of various genetic subtypes

AdipoRon, an AdipoR1 agonist [37], activates AdipoR1 signaling and is widely used in in vitro and in vivo experiments [36, 38–40] (Supplementary Fig. S1F). As AdipoR1 signaling activation enhances AMPK phosphorylation [41–43], we exposed the human and mouse cell lines to AdipoRon at different concentrations (10 or 40 µg/mL) and then analyzed AMPK phosphorylation using western blotting. AdipoRon treatment enhanced phosphorylated AMPK levels in all cell lines (Fig. 2C). Interestingly, after 24 h of drug exposure, no clear increase in AMPK phosphorylation was observed in MDA-MB-468 and 4T1-Luc cells in the high-concentration AdipoRon groups. As AMPK phosphorylation progresses minute by minute [44], we reasoned that phosphorylation had occurred at an earlier time point. Therefore, we analyzed AMPK phosphorylation 1 h after AdipoRon exposure. As expected, AMPK phosphorylation in MDA-MB-468 and 4T1-Luc cells was enhanced within 1 h of exposure to AdipoRon at a high concentration (Fig. 2C). These results indicate that the AdipoR1 receptor is universally expressed in breast cancer cell lines of different genetic backgrounds and that AdipoRon promotes AdipoR1 signaling activation in breast cancer cells.

### AdipoR1 stimulation inhibits breast cancer cell proliferation via apoptosis across cancer subtypes

**Fig. 3:**
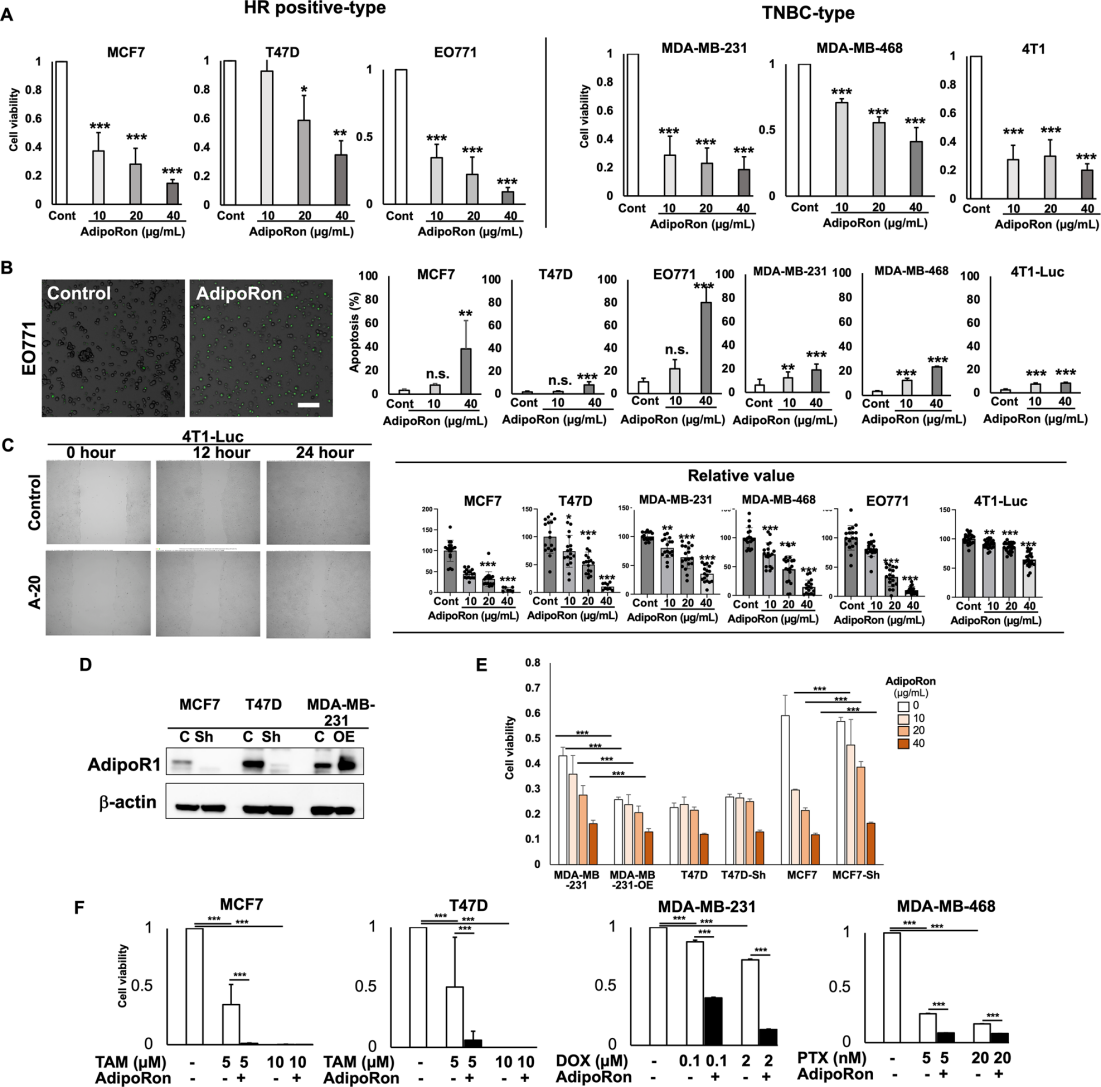
AdipoR1 stimulation suppresses cell proliferation and migration and induces apoptosis. **A** CCK-8 assay of human and mouse breast cancer cell lines after the indicated treatments. *P < 0.05, **P < 0.01, ***P < 0.001; one-way ANOVA followed by Tukey’s multiple comparison tests. **B** Apoptosis assay of breast cancer cell lines after the indicated treatments. Active caspase 3/7 protein was detected after 24 h of AdipoRon (10 or 40 µg/mL) or DMSO exposure (Cont). Left panel: representative image of EO771 cells treated or not with AdipoRon. Right panel: activated caspase 3/7 positivity after AdipoRon treatment in breast cancer cell lines. **P < 0.01, ***P < 0.001; one-way ANOVA followed by Dunnett’s multiple comparison tests. **C** Wound scratch assay of breast cancer cell lines after the indicated treatments. Left panel: representative images of 4T1-Luc cells after 0, 12, and 24 h in the presence or absence (control) of AdipoRon. Right panel: percentage wound recovery after AdipoRon treatment in breast cancer cell lines. *P < 0.05, **P < 0.01, ***P < 0.001; one-way ANOVA followed by Dunnett’s multiple comparison tests. **D** Western blot analysis of AdipoR1 expression in breast cancer cell lines. Sh cell lines stably expressing shRNA targeting AdipoR1, OE cell lines stably expressing AdipoR1 through lentiviral transfection. **E** CCK-8 assay of human and animal breast cancer cell lines stably expressing shRNA targeting AdipoR1 (Sh) or AdipoR1 (OE) after AdipoRon treatment. ***P < 0.001; one-way ANOVA followed by Dunnett’s multiple comparison tests. **F** CCK-8 assay of human breast cancer cell lines exposed to DMSO for control, AdipoRon (20 µg/mL), tamoxifen (TAM, 5 or 10 µM), paclitaxel (PTX, 5 or 20 nM), or doxorubicin (DOX, 0.1 or 2 µM). ***P < 0.001; one-way ANOVA followed by Tukey’s multiple comparison tests.

Based on the above results, we next scrutinized the phenotypic changes in cancer cells induced by AdipoRon. First, we examined the effect of AdipoRon treatment on proliferation. AdipoRon significantly inhibited proliferation in both HR-positive and TN cell lines in a concentration-dependent manner (Fig. 3A). To investigate whether apoptosis was involved, we examined caspase 3/7 activation using fluorescence staining (Fig. 3B). The results showed that AdipoRon exposure induced apoptosis via caspase 3/7 activation in all cell lines; however, the degree of activation differed among the cell lines (Fig. 3B): while apoptosis was significantly promoted in MCF7 and EO771 breast cancer cells, its induction in T47D and TN cells was limited. We next examined the effect of AdipoRon treatment on cell migration using a scratch assay. To avoid the effect of cell proliferation inhibition observed 48 h after drug exposure, we analyzed the cells within 24 h after AdipoR1 exposure. Migration was significantly reduced in all cell lines after AdipoRon treatment (Fig. 3C). These results demonstrated that the activation of AdipoR1 signaling by AdipoRon-induced apoptosis-mediated cell death and cell migration inhibition in breast cancer cells across genetic and molecular subtypes.

In pancreatic cancer cell lines, AdipoRon has been reported to induce mitochondrial uncoupling and increase glucose uptake, subsequently enhancing glycolysis. In addition, the glycolysis inhibitor 2-deoxy-D-glucose (2DG) was shown to enhance AdipoRon’s antiproliferative effects [45]. Based on these findings, we first examined the protein expression of UCP2, which is typically upregulated by mitochondrial uncoupling, as well as oxidative phosphorylation (OXPHOS)-related proteins and Tom20, which are known to decrease under mitochondrial dysfunction, in breast cancer cell lines treated with AdipoRon. No increase in UCP2 expression was observed in any of the cell lines. In contrast, MCF7 cells exhibited decreased expression of OXPHOS-related proteins, including SDHB and MTCO2, as well as Tom20 (Supplementary Fig. S2A). We then used hormone receptor-positive and triple-negative breast cancer cell lines to examine whether fluorescently labeled glucose, 2-(N-(7-nitrobenz-2-oxa-1,3-diazol-4-yl)amino)-2-deoxyglucose (2-NBDG), enhanced glucose uptake in response to AdipoRon treatment. We also evaluated whether 2DG enhanced the antiproliferative effect of AdipoRon. However, no significant enhancement was observed in either case (Supplementary Fig. S2B). Finally, UCP2 and Tom20 expression in breast cancer tissues from AdipoRon-treated mice was evaluated by immunohistochemistry, and no significant differences were observed compared with controls (Supplementary Fig. S2C). Taken together, these findings suggest that the metabolic alterations induced by AdipoRon may differ depending on the tumor origin and individual cell line.

### AdipoR1 expression correlates with the regulatory effect of AdipoR1 agonist on cell proliferation

To clarify whether cancer cell growth regulation by AdipoRon correlates with the amount of AdipoR1, we performed *ADIPOR1* expression regulation experiments. We generated *ADIPOR1* overexpression and suppression lines by transfecting human breast cancer cell lines with lentiviral *ADIPOR1* expression and shRNA vectors, respectively. AdipoR1 protein expression was increased and decreased in the *ADIPOR1* overexpression and suppression lines, respectively (Fig. 3D). The cell proliferation-inhibitory effect of AdipoRon treatment was significantly attenuated after *ADIPOR1* silencing and enhanced upon *ADIPOR1* overexpression (Fig. 3E). These results indicate that the AdipoR1 expression level correlates with the cell proliferation-regulatory effect of AdipoR1 agonist stimulation. On the other hand, in the correlation analysis of AdipoRon sensitivity and AdipoR1 mRNA expression in cancer cell lines from DepMap and the Human Protein Atlas, no significant correlation was observed between the inhibitory effect on cell proliferation and AdipoR1 (Supplementary Fig. S3). The AdipoRon concentration in DepMap was 2.5 µM (0.87 µg/mL), which is less than one-tenth of the concentration used in this study, suggesting that the difference in AdipoRon concentration may have influenced the results.

### AdipoRon and conventional therapy additively inhibit cancer cell proliferation

Next, we examined whether AdipoRon would enhance growth inhibition by conventional therapeutic agents in patients with breast cancer. HR-positive breast cancer cells (MCF7 and T47D) were concurrently treated with tamoxifen, a conventional therapeutic agent for HR-positive breast cancer, and AdipoRon; TN breast cancer cells (MDA-MB-231 and MDA-MB-468) were exposed to paclitaxel or doxorubicin, conventional TN breast cancer drugs, and AdipoRon. The combination treatments inhibited cell proliferation to a significantly greater extent than the single agents alone (Fig. 3F). Next, AdipoRon was administered in combination with a conventional therapeutic agent to cell lines with either silenced or overexpressed *ADIPOR1*. As a result, the combined inhibitory effect of the therapeutic agent and AdipoRon was significantly enhanced in *ADIPOR1*-overexpressing cells compared to control cells, whereas the therapeutic agent alone showed no such enhancement. Similarly, the combined inhibitory effect was significantly attenuated in *ADIPOR1*-silenced cells compared to control cells, even though the therapeutic agent alone did not show a significant attenuation (Supplementary Fig. S4). These results suggest that AdipoR1 agonists may contribute to breast cancer control when used in combination with standard breast cancer therapies.

### AdipoR1 stimulation markedly suppresses BRCA and ER protein expression in HR-positive breast cancer cell lines

**Fig. 4:**
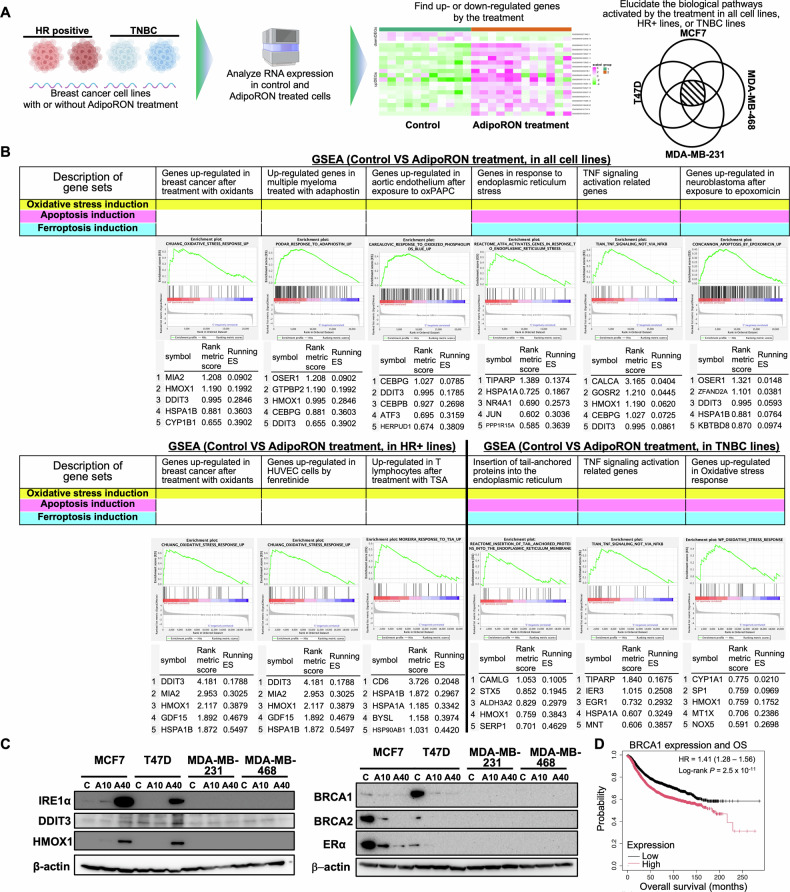
Gene expression analysis revealing that AdipoRon promotes oxidative stress, apoptosis, and ferroptosis in breast cancer cell lines. **A** Schematic representation of RNA expression analysis of control (DMSO) and AdipoRon (40 µg/mL)-treated breast cancer cell lines. **B** Gene set enrichment analysis (GSEA) of AdipoRon-treated breast cancer cell lines. GSEA plots for multiple gene sets involved in oxidative stress, apoptosis, and ferroptosis that were upregulated by AdipoRon treatment are shown. **C** Western blot analysis of proteins up- or downregulated by AdipoRon treatment. C control (DMSO), A10 10 µg/mL AdipoRon, A40 40 µg/mL AdipoRon. **D** Kaplan–Meier analysis of the relationship between *BRCA1*-high or *BRCA1*-low expression in breast cancer and overall survival. The association between clinical parameters and survival was determined using univariate Cox regression; *n* = 4929.

The above results showed that AdipoR1 stimulation suppresses the growth of HR-positive and TN breast cancer cells. However, the shared mechanisms of AdipoR1-mediated cell growth inhibition across different breast cancer cell lines, as well as the mechanisms specific to each molecular subtype, remained unclear. Therefore, to examine the common and unique mechanisms, we collected RNA from four breast cancer cell lines treated with AdipoRon and performed RNA sequencing (RNA-seq) (Fig. 4A). Gene set enrichment analysis (GSEA) revealed that gene sets related to the induction of oxidative stress, apoptosis, and ferroptosis were enriched after AdipoRon treatment (Fig. 4B). Notably, mRNA levels of the ferroptosis genes *DDIT3*, *HMOX1*, and *ERN1* were significantly increased upon AdipoRon exposure in all four breast cancer cell lines. Western blotting showed that the corresponding proteins (DDIT3, HMOX1, and IRE1α (encoded by *ERN1*)) were also upregulated in MCF7 and T47D cells after exposure to AdipoRon (Fig. 4C). Ferroptosis marker (lipid peroxide) positivity was significantly increased in MCF7 cells after exposure to AdipoRon (Supplementary Fig. S5A).

The GSEA results showed that genes related to lipid synthesis and insulin resistance were enriched in the HR-positive breast cancer cell lines, whereas in TN breast cancer cell lines, gene sets involved in various pathways, including necrosis, extracellular vesicle transport, and suppression of myelodysplastic syndrome, were enriched (Supplementary Fig. S5B).

We next examined changes in the expression of genes important for breast cancer cell proliferation based on the RNA-seq results. *BRCA1* and *BRCA2* encode DNA repair enzymes, and increased expression of these genes in cancer cells has been implicated in cancer survival [46, 47]. *BRCA1* and *BRCA2* mRNA expression in HR-positive breast cancer cell lines was decreased after AdipoRon administration (Supplementary Fig. S5C). Western blot results showed that AdipoRon exposure significantly suppressed BRCA2 and BRCA1 protein expression in MCF7 and T47D cells, respectively (Fig. 4C). BRCA1 protein was also suppressed in MDA-MB-231 cells (Supplementary Fig. S5C). Increased BRCA2 expression has been associated with poor breast cancer prognosis (Fig. 4D). The ER controls cell proliferation in HR-positive breast cancer, which accounts for 60%–70% of breast cancer cases [48]. The RNA-seq data showed a decrease in *ESR1* mRNA expression in HR-positive breast cancer cell lines (Supplementary Fig. S5C). AdipoR1 stimulation significantly suppressed ER protein expression in both MCF7 and T47D cells (Fig. 4C). TROP2 is overexpressed in many cancers, including breast cancer, and silencing TROP2 expression suppresses cell proliferation and promotes apoptosis [49, 50]. AdipoR1 stimulation significantly suppressed TROP2 protein and mRNA expression in T47D cells (Supplementary Fig. S5D). In addition, TROP2 mRNA was suppressed by AdipoR1 stimulation in one TN breast cancer cell line, MDA-MB468 (Supplementary Fig. S5D). These results suggest that, at least in HR-positive breast cancer, AdipoR1 is involved in the activation of ferroptosis and apoptosis, alters the expression of DNA repair enzymes, and suppresses ER expression, thus suppressing cell proliferation. These diverse mechanisms of cancer proliferation inhibition may also have contributed to the additive suppressive effect of AdipoRon and existing drugs in the various breast cancer cell lines.

### AdipoR1 stimulation inhibits tumor growth and induces necrosis in vivo

**Fig. 5:**
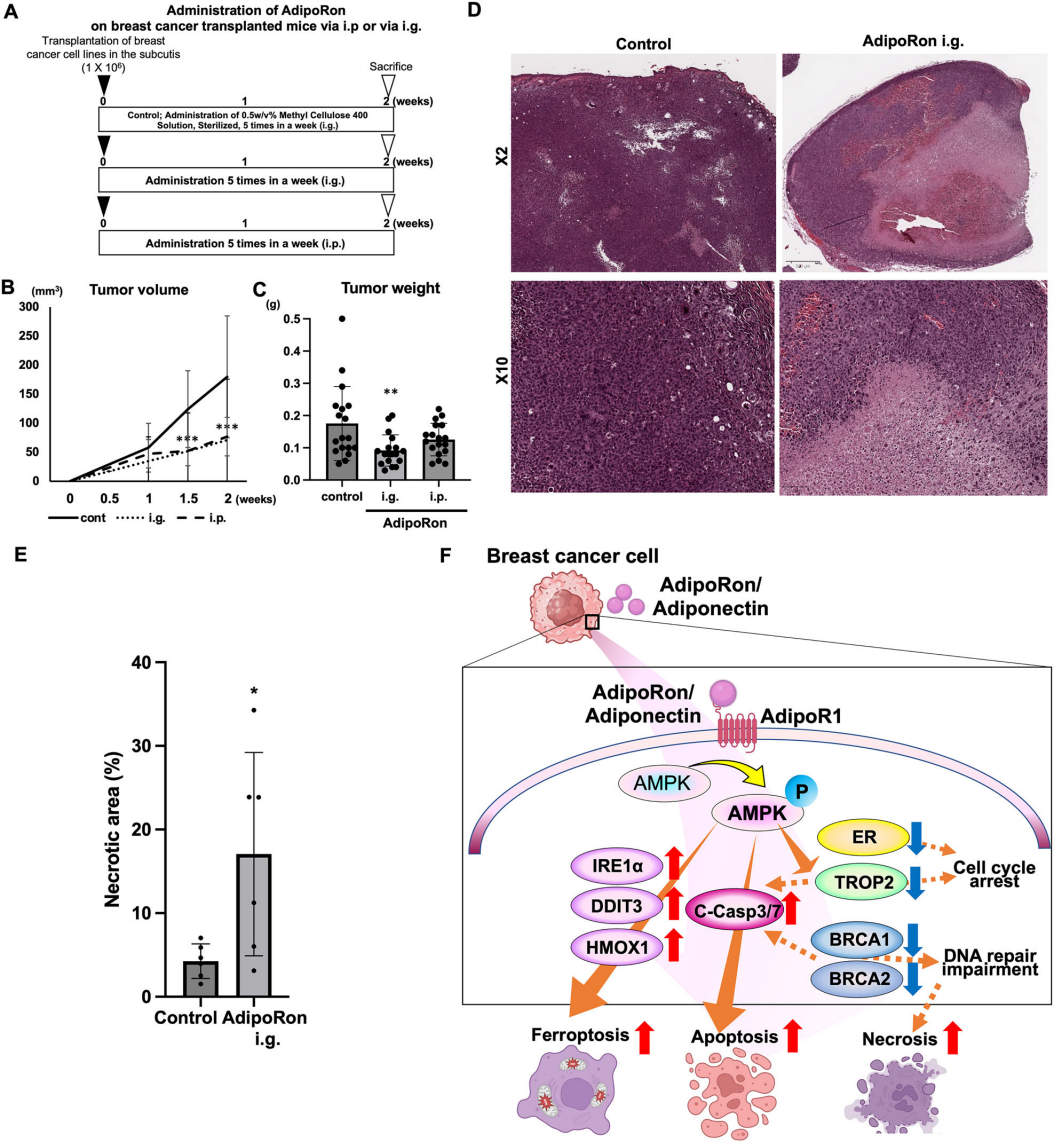
Adiponectin receptor stimulation attenuates breast tumor growth in vivo. **A** Schematic representation of the animal experiment. EO771 breast cancer cells were transplanted into the subcutis of C57BL6 mice. The dose of AdipoRon was 50 mg/kg. **B** Tumor volumes of EO771 transplants with or without AdipoRon treatment. ***P < 0.001; one-way ANOVA followed by Dunnett’s multiple comparison tests. **C** Tumor weight of EO771 transplants with or without AdipoRon treatment. **P < 0.01; one-way ANOVA followed by Dunnett’s multiple comparison tests. **D** Representative images of untreated (left) and AdipoRon-treated (right) breast cancer cell tumors. Upper images: scale bar = 500 µm; lower images: scale bar = 100 µm. **E** Necrotic area of EO771 transplants with or without AdipoRon treatment. *P < 0.05; one-way ANOVA followed by an unpaired two-tailed Student’s *t*-test. **F** Model of AdipoR1 signaling-induced cell death pathway. AdipoRon/Adiponectin activates AdipoR1 signaling and phosphorylates downstream AMPK, upregulating cell death-related proteins (DDIT3, HMOX1, IRE1α, and cleaved caspase 3/7) and downregulating survival-related proteins (ER, TROP2, BRCA1, and BRCA2). These proteins promote apoptosis, ferroptosis, and necrosis, either independently or synergistically.

The above experiments revealed that AdipoRon activates a multifaceted cancer growth suppression mechanism in HR-positive breast cancer. Therefore, we investigated the tumor-suppressive effect of AdipoR1 stimulation in vivo using mice with HR-positive breast cancer (EO771) xenografts. When AdipoRon was orally administered to mice with mouse HR-positive tumors (Fig. 5A), tumor growth was significantly inhibited (Fig. 5B, C). Histopathological results revealed massive tumor necrosis after AdipoRon treatment (Fig. 5D). Statistical analysis showed the percentage of necrotic area in tumors in the group orally administered AdipoRon was significantly higher compared to that in the control group (Fig. 5E). No side effects of AdipoRon administration were observed in the mice’s behavior, such as reduced activity. Additionally, we did not observe any diarrhea, skin abnormalities, or paleness of the tail or ears. During the administration period, the AdipoRon-treated group showed no significant weight gain or loss compared to the control group (Supplementary Fig. S6A). Furthermore, histological analysis of the liver revealed no significant changes compared to the control group, and no findings related to anemia, such as erythroblast clusters suggesting extramedullary hematopoiesis, were observed (Supplementary Fig. S6B). Furthermore, immunostaining of liver tissue from the AdipoRon-treated and control groups for EPO and HIF2a, indicators of anemia, revealed no significant differences in the percentage or intensity of stained cells between the control and AdipoRon-treated groups (Supplementary Fig. S6C).

## Discussion

In this study, we found that AdipoR1 was highly expressed in breast cancer across molecular subtypes, and its signaling activation triggered multiple cell death pathways, including apoptosis, ferroptosis, and necrosis via the regulation of multiple protein expression profiles (Fig. 5F). We demonstrated that AdipoR1 stimulation significantly suppressed the proliferation of breast cancer cells, regardless of the genetic or molecular subtype, and enhances the expression of apoptosis- and ferroptosis-related genes in HR-positive breast cancer. Further, we demonstrated that AdipoR1 stimulation controls ER, TROP2, and BRCA expression. The in vivo study showed that AdipoR1 stimulation induced necrosis. Overall, AdipoR1 stimulation appears to promote at least three different cell death pathways. Our findings suggest that the antitumor mechanism of AdipoR1 stimulation differs from that of conventional therapies and may offer additive benefits when used in combination, as shown in our in vitro study.

Gene expression analysis using public data revealed that the expression of multiple adipokines, including adiponectin, is altered in certain cancers. In particular, in breast cancer, AdipoR1 expression is significantly enhanced, as indicated by analyses using multiple datasets and multiple controls (normal tissues or diploids). These results indicate that adipokine-targeted cancer therapy should be limited to cancer types in which adipokine signaling is deemed suitable for targeting based on AdipoR1 expression. In the future, we plan to investigate the significance of other adipokine receptors that are significantly differentially expressed in cancers as potential therapeutic targets.

Experiments using four breast cancer cell lines of different genetic subtypes confirmed AdipoR1 mRNA and protein expression in all cell lines, and AdipoRon treatment was found to enhance AMPK phosphorylation and significantly inhibit proliferation. Together, these results suggest that AdipoR1 stimulation may have a universal antitumor effect on breast cancers that express AdipoR1, regardless of the breast cancer genotype and subtype.

We examined the correlation between AdipoRon sensitivity and AdipoR1 mRNA expression in all 527 cancer cell lines and 13 breast cancer cell lines published in DepMap and the Human Protein Atlas. Contrary to our results, no significant correlation was found, and the antiproliferative effects were unclear. According to the DepMap data, the AdipoRon concentration used in drug-sensitivity testing of cell lines was 2.5 µM (0.87 µg/mL). In our cell experiments, AdipoRon concentrations of 10, 20, and 40 µg/mL were used. Significant antiproliferative effects were observed in all breast cancer cell lines at concentrations >20 µg/mL. However, concentrations <10 µg/mL did not activate AdipoR1 signaling, as indicated by AMPK phosphorylation, or significantly inhibit proliferation in some cell lines (Figs. 2 and 3). Furthermore, in studies by other groups, significant cancer growth inhibition by AdipoRon has been observed at concentrations similar to or higher than those in this study (20 µg/mL), with no significant inhibitory effect observed at concentrations below 10 µg/mL [38, 39, 51]. Therefore, the lack of correlation between AdipoR1 and the effects of AdipoRon in cell lines in the DepMap data, and the unclear cell growth inhibition effect, may be due to differences in the AdipoRon concentrations used to treat cell lines between DepMap and this study.

Hence, it is necessary to examine a larger number of cancer cell lines at the dose used in this study and to examine the correlation between AdipoR1 expression and AdipoRon sensitivity in more detail.

AdipoRon and breast cancer drugs inhibited cancer cell proliferation in an additive manner. In vivo, the antitumor action of AdipoRon alone was limited. The gene expression analysis results indicate that AdipoRon has a different mechanism of action than existing anticancer drugs; therefore, combination therapies have strong potential for clinical applications.

When we examined gene expression changes after exposure to AdipoRon in breast cancer cell lines of different subtypes, we found that gene sets related to oxidative stress, apoptosis, and ferroptosis induction were upregulated in all cell lines, suggesting that cell proliferation control is a common mechanism of AdipoRon across breast cancer subtypes. However, further analysis revealed that ferroptosis-inducing protein expression was enhanced only in HR-positive breast cancers, suggesting that AdipoRon mechanisms involving post-transcriptional modifications may differ among breast cancer subtypes. Therefore, factors other than ferroptosis may be involved in cell proliferation inhibition in TN breast cancers.

To our knowledge, this study shows for the first time that AdipoR1 stimulation downregulates BRCA gene and protein expression in HR-positive breast cancer cells. Wild-type MDA-MB-231, MDA-MB-468, MCF7 [52], and T47D [53] cells do not harbor *BRCA1* and *BRCA2* mutations. As shown in Fig. 4D and in a previous report [46], high *BRCA1* expression is a poor prognostic factor for breast cancer. Although studies have reported that cell proliferation inhibition leads to high *BRCA1* expression in cancer [54–56], considering that BRCA1 is involved in homologous recombination repair [57, 58], high BRCA1 expression may lead to treatment resistance, and inhibition of BRCA expression may represent a new breast cancer treatment modality. In particular, the combination of AdipoR1 stimulators and PARP inhibitors may be a promising effective treatment for breast cancer patients with no BRCA mutations and high BRCA mRNA levels. Further research is needed to unravel how the regulation of BRCA1 and BRCA2 expression is involved in breast cancer proliferation.

AdipoRon suppresses ER expression in HR-positive breast cancer cell lines, and ER signaling is the main proliferation-enhancing pathway in HR-positive breast cancer [59, 60]. AdipoRon exerted additive effects with hormonal drugs in vitro and may therefore have therapeutic effects on HR-positive breast cancers that have become resistant to conventional hormonal drugs.

Stimulation of AdipoR1 by AdipoRon reduced TROP2 expression. TROP2 is widely known as a target of antibody-drug conjugates (ADCs); it is widely expressed in breast cancer [61] and is involved in cell proliferation [49, 50]. To expand target therapy not only in HR-positive breast cancer but also in TROP2-positive TN cancer, control of TROP2 expression by AdipoR1 stimulation is important for expanding the application of adiponectin signaling.

A previous study [45] in pancreatic cancer cell lines reported that AdipoRon induces mitochondrial uncoupling, decreases ATP levels, and enhances glucose uptake, and that the glycolysis inhibitor 2-deoxy-D-glucose (2DG) further enhances its antitumor effects. To determine whether these findings also apply to breast cancer cells, we analyzed the expression of UCP2, which is induced by mitochondrial uncoupling, as well as OXPHOS-related proteins and Tom20, which decrease under mitochondrial dysfunction. However, we did not observe consistent evidence of mitochondrial uncoupling in the breast cancer cell lines tested, suggesting that AdipoRon-induced mitochondrial dysfunction may be cell line-specific. We further examined the effect of AdipoRon on glucose uptake using a 2-(N-(7-nitrobenz-2-oxa-1,3-diazol-4-yl)amino)-2-deoxyglucose (2-NBDG) assay in hormone receptor-positive and triple-negative breast cancer cell lines and assessed whether 2DG could enhance AdipoRon’s antiproliferative effect. No significant enhancement was observed in either cell line. These results suggest that the metabolic effects of AdipoRon and their potentiation by glycolysis inhibitors may depend on the metabolic characteristics of the tumor type and individual cell lines. Further studies are warranted to examine in more detail the regulation of mitochondrial function by AdipoRon in breast cancer cells, including measurement of oxygen consumption rates using a Seahorse Bioanalyzer in collaborative experiments.

In the animal experiment, immunocompetent mice were transplanted with mouse-derived breast cancer cells to examine the effects of AdipoRon treatment on tumor growth. In the future, we plan to conduct animal experiments using human breast cancer cell lines to examine the effects of AdipoR1 signaling activation on human breast cancer cells in vivo. In this in vivo experiment, we administered AdipoRon early, before the transplanted tumors reached a certain size, because AdipoRon’s inhibitory effect on cancer cell growth was limited in vitro (Fig. 3). It is generally known that the efficacy of anticancer drugs is reduced in vivo compared with in vitro due to differences in drug solubility in culture medium and blood [62], as well as factors such as metabolism and the tumor microenvironment in vivo [63]. Given the insufficient tumor-suppressive effect observed in vitro, we anticipated that the in vivo effects of AdipoRon would also be limited. Early drug administration is advantageous for assessing the effects on early tumor growth and on tumor cells before the microenvironment is fully established in vivo [64]. Therefore, we chose this drug administration schedule. However, because many patients present with advanced cancers in clinical settings, further animal experiments using larger tumors are necessary. Moreover, as AdipoRon alone is expected to have a limited antitumor effect, we plan to conduct combination studies with other anticancer drugs. A comprehensive search of AdipoR1 expression in normal tissues using the Genotype-Tissue Expression (GTEx) project [65] and the Human Protein Atlas revealed that AdipoR1 is expressed in hematopoietic and epidermal cells, suggesting that AdipoR1 stimulation by AdipoRon may lead to side effects such as anemia or skin abnormalities. In this experiment, mice treated with AdipoRon did not show reduced activity or pale tail coloration associated with anemia compared with control mice. No skin abnormalities, significant weight loss (Supplementary Data), or gastrointestinal side effects such as diarrhea were observed. Histological examination also revealed no notable morphological abnormalities or significant increases in the expression of anemia-related marker proteins, including EPO and HIF-2α [66, 67], in the liver compared with controls. Although no significant adverse effects of AdipoRon administration have been reported in mice [37], the scope of previous studies and our own assessment is limited, and the possibility of unrecognized side effects cannot be excluded. In addition, no clinical trials involving AdipoRon have been registered in the United States, Europe, or Japan, based on searches of ClinicalTrials.gov, the EU Clinical Trials Register, the UMIN Clinical Trials Registry (UMIN-CTR), and PubMed. Therefore, the potential side effects of AdipoRon in humans must be carefully evaluated through further preclinical studies, particularly in the context of developing therapeutics targeting the adiponectin receptor. AdipoR1 signaling was activated using the small molecular compound AdipoRon. Small-molecule compounds may exert unexpected off-target effects. Ideally, AdipoR1 signaling is activated using synthetic adiponectin; however, in vivo, adiponectin exists as dimers, trimers, and hexamers [68–70], which have vigorous physiological activity, and synthesizing polymers is challenging. Available synthetic adiponectin compounds are monomers and have low physiological activity. In the future, we plan to synthesize high-molecular-weight adiponectin in collaboration with academic research institutes and companies to investigate its cancer-suppressive effects.

## Conclusion

Our findings identify AdipoR1 activation as a promising multimodal therapeutic strategy for breast cancer across molecular subtypes, as it integrates metabolic stress signaling, cell death pathways, DNA repair, and hormone receptor suppression. The broad efficacy of this approach may provide additional therapeutic benefits to patients with AdipoR1-positive breast cancer at all stages, regardless of hormone receptor and HER2 status, which are current factors in the treatment decision tree. Further preclinical studies are required for clinical application, and screening for more potent AdipoR1 agonists is also necessary. We plan to conduct retrospective and prospective non-interventional studies of blood adiponectin levels and AdipoR expression in breast cancer patients to examine whether prognosis, treatment resistance, and metastasis differ between breast cancer patients with high blood adiponectin levels and AdipoR expression and those with the opposite phenotype.

## Materials and methods

Detailed information is provided in the Supplementary Information.

### Cell lines and constructs

MCF7, T47D, MDA-MB-231, MDA-MB-468, EO771, and 4T1 cells were purchased from the American Type Culture Collection (ATCC, Manassas, VA, USA). MDA-MB-231-Luc and 4T1-Luc cells were procured from the Japan Collection of Research Bioresources Cell Bank (JCRB, Tokyo, Japan). Lentiviral shRNA constructs targeting *ADIPOR1* were obtained from Sigma-Aldrich (St. Louis, MO, USA). A stable *ADIPOR1* overexpression construct was generated by cloning the open reading frame of *ADIPOR1* downstream of the CMV promoter into a pLOC lentiviral vector purchased from Horizon Discovery (Cambridge, UK). All cell lines used in this study were authenticated within the past year using short tandem repeat (STR) profiling by BEX (Tokyo, Japan) and were tested for mycoplasma contamination by PCR (e-Myco™ plus Mycoplasma PCR Detection Kit, #25237, South Korea). All experiments were conducted using mycoplasma-free cells. All in vitro experiments were repeated at least three times independently, and all plots show the mean of ≥3 technical replicates per condition for each biological replicate (n), obtained from ≥3 independent experiments.

### Adipokine signaling-related gene expression analysis across cancer types

In total, 37 genes encoding adipose secretory factors, including adipokines and their receptors, were analyzed (Table 1). The mRNA expression and gene amplification or deletion of these genes were analyzed in 31 cancer types using data from 8433 patients in the TCGA database (https://www.cancer.gov/ccg/research/genome-sequencing/tcga?form=MG0AV3), an open-access database available at cBioPortal (https://www.cbioportal.org) [71–73]. We also performed similar analyses using TCGA data for breast cancer (*n* = 994) and data from the Molecular Taxonomy of Breast Cancer International Consortium (METABRIC) database (*n* = 1604).

### Western blot analysis

The proteins extracted from cells were loaded onto and separated on sodium dodecyl sulfate polyacrylamide gels and transferred to nitrocellulose membranes. The membranes were incubated overnight at 4 °C with the following rabbit monoclonal primary antibodies: AdipoR1 and TROP2 (Abcam, Cambridge, UK; ab50675 and ab227691); AMPKα, phospho-AMPKα, β-actin, IRE1α, CHOP, HO-1, BRCA1, BRCA2, ERα, and cleaved caspase 3 (Cell Signaling Technology, Danvers, MA, USA; 5831S, 2535S, 4970S, 3294S, 2895S, 43996S, 14823S, 10741S, 8644S, and 9664S, respectively). The blots were incubated with HRP-conjugated goat anti-rabbit antibody (Cytiva, Marlborough, MA, USA) for one hour, visualized by ECL (Cytiva, Marlborough, MA, USA), and imaged with a Chemidoc Touch MP (Bio-Rad, Hercules, CA, USA). All experiments were repeated at least three times independently.

### Immunohistochemistry (IHC) using tissue microarray cores

Tissue microarrays (TMAs) prepared from anonymized paraffin-embedded tissue blocks of breast cancer from the included patients were used for immunostaining. In brief, TMA blocks were sectioned at 5-μm thickness. The sections were incubated with antibodies against AdipoR1 (ab70362, Abcam) at room temperature (20–25 °C) for 1 h. After washing with PBS, the sections were incubated with species-specific secondary antibodies (724132, 724142; Nichirei Biosciences), washed, and incubated with 0.5 mg/mL 3,3′-diaminobenzidine (725191; Nichirei Biosciences) for visualization.

### Apoptosis assay

Apoptosis was assessed using a cleaved caspase 3/7 detection kit (Invitrogen). After exposing cells to the kit reagent for 30 min, green fluorescence-stained cells were counted using a Countess 3 FL automated cell counter (Thermo Fisher Scientific). The assay was repeated three times independently, and the average values were calculated.

### Ferroptosis assay

Ferroptosis was assessed using a Liperfluo ferroptosis detection kit (Cat# L248; Dojindo). Trypsinized cells were incubated with Liperfluo in an incubator for 1 h. Then, the cells were washed with Hank’s balanced salt solution (Cat# 14025092; Thermo Fisher Scientific), and green fluorescence-stained cells were counted using the Countess 3 FL automated cell counter. The assay was repeated three times independently, and the average values were calculated. Data are shown as mean ± SD from at least two independent experiments with duplicate or triplicate samples.

### In vivo experiments

All animal experiments conformed to the ARRIVE guidelines and were approved by the Kanagawa Cancer Center Animal Experimentation Committee (approval number 01-07).

The sample size for the animal experiments was determined using GraphPad Prism. For the planned three-group comparison, a one-way ANOVA was used with a standardized effect size of 0.65, a power of 0.8, and a two-tailed alpha of 0.05. Based on this calculation, nine animals per group were required, and the experiments were conducted accordingly.

Ten-week-old female C57BL6 (Jackson Laboratory Japan, Yokohama, Japan) mice were subcutaneously implanted with 1 × 10^6^ EO771 cells on the left and right flanks. Mice in the control group received 0.4% cellulose, whereas those in the treatment group received 50 mg/kg AdipoRon (Adipogen) i.p. or i.g., five times a week for two weeks. Tumor diameters were measured twice a week during the treatment. Two weeks after treatment, the mice were sacrificed, and the tumors were excised and weighed. The experiment was performed independently three times with *n* = 3 mice in each of the control, i.p, and i.g groups. Animals were randomly assigned to groups without weight bias.

### RNA-sequencing (RNA-seq) analysis

Human breast cancer cells (MCF7, T47D, MDA-MB-231, and MDA-MB-468) were exposed to AdipoRon at 10 µg/mL or 40 µg/mL or to dimethyl sulfoxide (DMSO; Cat# 043-07216; Fujifilm Wako) as a control. After 24 h of exposure, RNA was extracted from all cells using an RNeasy Mini kit (Qiagen). RNA quality was assessed using a Nanodrop (Thermo Fisher). Sequencing libraries were prepared from qualified total RNA using the SMARTer Stranded Total RNA Sample Prep Kit (635005; Takara Bio) and sequenced using the Illumina NextSeq500 (Illumina), generating 236-bp reads. RNA-seq was outsourced to Takara Bio. Differentially expressed genes were identified based on *P* < 0.05 (*t*-test) and log2(fold change) >1 and functionally annotated using Gene Ontology and Kyoto Encyclopedia of Genes and Genomes pathway enrichment analysis (KEGG). Ingenuity Pathway Analysis (IPA, Qiagen, Hilden, Germany) was conducted using our original datasets. The sequence reads produced in this study are deposited at Gene Expression Omnibus (GEO) under accession No. GSE290491.

### In silico analysis using public data

In silico analysis was performed using data from cBioPortal, the Cancer Cell Line Encyclopedia (CCLE; https://sites.broadinstitute.org/ccle/), and the Human Protein Atlas. The mRNA expression and gene amplification/deletion of 37 adipokines and adipokine receptors were analyzed using cBioPortal, based on RNA-seq data of tumor tissues from cancers originating in various organs. AdipoR1 expression status in the various cancer types was analyzed using the search tool of the Human Protein Atlas. ER, PgR, HER2, and AdipoR1 mRNA expression in breast cancer cell lines was analyzed using the CCLE website. No computer code was used in this study.

### Lentiviral transfection

Stable gene knockdown or expression was achieved using the ViraPower Lentiviral expression system (Invitrogen) according to the manufacturer’s protocol. Gene manipulation from polyclonal stable cell lines was checked using western blotting. Lentiviral shRNA constructs targeting *ADIPOR1* were obtained from Sigma-Aldrich. Stable *ADIPOR1* overexpression was achieved by cloning the open reading frame of *ADIPOR1* downstream of the CMV promoter in the pLOC lentiviral vector (Dharmacon).

### Statistical analysis

Data are reported as mean ± SD and were analyzed using GraphPad Prism 7.0 software (SCR_002798, GraphPad Software, La Jolla, CA, USA). Means of the two groups were compared using Student’s unpaired *t*-test. For multiple group comparisons, an ordinary ANOVA was employed, followed by the Tukey–Kramer or Dunnett’s multiple comparison tests. Statistical significance was set to *P* < 0.05. Pearson’s chi-squared test was used to determine correlations between clinical parameters and overall survival. In Ingenuity Pathway Analysis (SCR_008653, Qiagen), canonical pathway activation was assessed based on the z-score, with a positive value indicating pathway activation and a negative value indicating pathway inhibition. Kaplan–Meier plots were generated using the Kaplan–Meier plotter [74].

### Ethical approval

This study was approved by the Kanagawa Cancer Center Ethics Committee (approval number: 2020 EKI-37) and conducted in accordance with the study protocol approved by the center. Comprehensive written informed consent was obtained from all patients included in the study. All animal experiments conformed to the ARRIVE guidelines and were approved by the Kanagawa Cancer Center Animal Experimentation Committee (approval number 01-07). All methods, including study design, sample size, inclusion and exclusion criteria, outcome measures, method of euthanasia, timing of tissue collection after euthanasia, and statistical methods, were performed according to relevant guidelines and regulations.

## Supplementary information

Supplementary Information: Detailed Materials and Methods

Supplementary Figure S1

Supplementary Figure S2

Supplementary Figure S3

Supplementary Figure S4

Supplementary Figure S5

Supplementary Figure S6

all raw_western_blot_images_of_main_figures

all_raw_western_blot_images_of_supplementary_figures

## Data Availability

Detailed information is provided in the Supplementary Information.
