# Supplementary material for: AdipoR1–AMPK axis suppresses breast cancer across molecular subtypes via multimodal cell death pathways, including ferroptosis and apoptosis
Source: Cell Death Dis. 2026 Mar 26;17(1):384. doi: 10.1038/s41419-026-08583-7 (PMC13049035; doi:10.1038/s41419-026-08583-7)
Supplement: Supplementary file 1 — Supplementary Information: Detailed Materials and Methods [file 41419_2026_8583_MOESM1_ESM.docx]

**Supplementary Information**

**Detailed materials and methods**

***Cell lines and constructs***

MCF7 (Cat# HTB-22, RRID: CVCL_0031), T47D (Cat# HTB-133, RRID: CVCL_0553), MDA-MB-231 (Cat# CRM-HTB-26, RRID: CVCL_0062), MDA-MB-468 (Cat# CRM-HTB-132, RRID: CVCL_0419), EO771 (Cat# CRL-3461, , RRID: CVCL_GR23), and 4T1 (Cat# CRL-2539, RRID: CVCL_0125) cells were purchased from the American Type Culture Collection (ATCC, Manassas, VA, USA). MDA-MB-231-Luc (Cat# 1559) and 4T1-Luc (Cat# 1447, RRID: CVCL_E3GH) cells were procured from the Japan Collection of Research Bioresources Cell Bank (JCRB, Tokyo, Japan). The cells were cultured in RPMI1640 medium (Cat# 189-02025; Fujifilm Wako, Tokyo, Japan) supplemented with 10% fetal bovine serum (FBS; Cat# A5256701, Gibco/Thermo Fisher Scientific, Waltham, MA, USA) and 1% antibiotic-antimycotic (100X; Cat# 15240062; Gibco/Thermo Fisher Scientific) in 100-mm tissue culture plates at 37°C in the presence of 5% CO_2_. The medium was renewed every 3 days, and the cells were split every 4 days. Stable gene knockdown or expression was achieved using the ViraPower Lentiviral Expression System (Cat# K497500; Thermo Fisher Scientific), according to the manufacturer’s protocol. Gene manipulation in stable polyclonal cell lines was checked using western blotting, and cells were passaged for no longer than a month for use in experiments. Cells were periodically checked for *Mycoplasma* contamination. Lentiviral shRNA constructs (MISSION™ TRC shRNA, TRCN0000063045 and TRCN0000299279) targeting *ADIPOR1* were obtained from Sigma-Aldrich (St. Louis, MO, USA). A stable *ADIPOR1* overexpression construct was constructed by cloning the open reading frame of *ADIPOR1* downstream of the CMV promoter into a pLOC lentiviral vector purchased from Horizon Discovery (Cambridge, UK). All cell lines used in this study were authenticated within the past year using short tandem repeat (STR) profiling by BEX (Tokyo, Japan) and were tested for mycoplasma contamination by PCR (e-Myco™ plus Mycoplasma PCR Detection Kit, #25237, South Korea). All experiments were conducted using mycoplasma-free cells.

***Adipokine signaling-related gene expression analysis across cancer types***

In total, 37 genes encoding adipose secretory factors, including adipokines and their receptors were analyzed (Table 1). The mRNA expression and gene amplification or deletion of these genes were analyzed in 31 cancer types (acute myeloid leukemia, adrenocortical carcinoma, bladder urothelial carcinoma, brain lower grade glioma, breast invasive carcinoma, cervical squamous cell carcinoma, cholangiocarcinoma, colorectal adenocarcinoma, diffuse large b-cell lymphoma, esophageal adenocarcinoma, glioblastoma multiforme, head and neck squamous cell carcinoma, kidney chromophobe carcinoma, kidney renal clear cell carcinoma, kidney renal papillary cell carcinoma, liver hepatocellular carcinoma, lung adenocarcinoma, lung squamous cell carcinoma, mesothelioma, ovarian serous cystadenocarcinoma, pancreatic adenocarcinoma, pheochromocytoma and paraganglioma, prostate adenocarcinoma, sarcoma, skin cutaneous melanoma, stomach adenocarcinoma, testicular germ cell tumors, thymoma, thyroid carcinoma, uterine carcinosarcoma, uterine corpus endometrial carcinoma, and uveal melanoma) using data from 8,433 patients in the TCGA database (https://www.cancer.gov/ccg/research/genome-sequencing/tcga?form=MG0AV3), an open-access database available at cBioportal (https://www.cbioportal.org) ^63, 64, 65^. We also performed similar analyses using TCGA data for breast cancer (*n* = 994) and data from the Molecular Taxonomy of Breast Cancer International Consortium (METABRIC) database (*n* = 1,604). As controls, normal samples were used for TCGA database analysis, and all samples for METABRIC database analysis.

***Western blot analysis***

Proteins were extracted from MCF7, T47D, MDA-MB-231, MDA-MB-468, EO771, and 4T1-Luc cells using RIPA buffer (FUJIFILM Wako Pure Chemical Corporation, Tokyo, Japan) containing protease inhibitor (cOmplete Mini, Roche, Basel, Switzerland) and phosphatase inhibitor (PhosphoSTOP, Roche). The proteins (20 μg) were loaded onto and separated in sodium dodecyl sulfate polyacrylamide gels and transferred to nitrocellulose membranes.

The membranes were incubated overnight at 4°C with the following rabbit monoclonal primary antibodies: AdipoR1 and TROP2 (Abcam, Cambridge, UK; ab50675 and ab227691); AMPKα, phospho-AMPKα, β-actin, IRE1α, CHOP, HO-1, BRCA1, BRCA2, ERα, cleaved caspase-3, Tom20, and UCP2 (Cell Signaling Technology, Danvers, MA, USA; 5831S, 2535S, 4970S, 3294S, 2895S, 43996S, 14823S, 10741S, 8644S, 9664S, 42406S, and 89326S, respectively) and mouse monoclonal antibodies: total OXPHOS cocktail (Abcam; ab110411) including NDFUB8, SDHB, UQCRC2, MTCO2, and ATP5A (Abcam; ab110242, ab14714, ab14745, ab110258, and ab14748, respectively). The blots were washed and incubated with horseradish peroxidase-conjugated goat anti-rabbit secondary antibody (Cytiva, Marlborough, MA) for 1 h and visualized using enhanced chemiluminescence using ECL Western blotting detection reagent (Cytiva). Protein bands were imaged with a Chemidoc Touch MP Imaging System (Bio-Rad, Hercules, CA, USA).

***Cell Counting Kit-8 (CCK-8) assay***

Cells of breast cancer cell lines were seeded in 96-well plates and incubated in an FBS-free culture medium for 24 h for cell cycle synchronization. Then, the cells were exposed to 10, 20, or 40 µg/mL of AdipoRon (Cat# SML0998; Sigma-Aldrich), 5 or 10 µM 4-hydroxytamoxifen (Cat# H6278; Sigma-Aldrich), 0.1 or 2 µM doxorubicin hydrochloride (Cat# 15007; Cayman Chemical), and 5 or 20 nM paclitaxel (Cat# 10461; Cayman Chemical) for 48 h or 72h, and cell viability was measured using a CCK-8 assay (Cat# 347-07621; Dojindo Laboratories, Kumamoto, Japan). The optical density at 450 nm in sample and control wells was measured using a multimode plate reader (EnSpire Alpha 2390; PerkinElmer, Waltham, MA, USA). The data are shown as mean ± standard deviation (SD) from at least two independent experiments with duplicate or triplicate samples.

***2-(N-(7-Nitrobenz-2-oxa-1,3-diazol-4-yl)amino)-2-deoxyglucose (2-NBDG) assay***

For the 2-NBDG uptake assay, we followed the protocol described in the previous report (PMID12345). Cells were treated with AdipoRon (25 or 50  µM) for 48 h in 6-well plates. The medium was then replaced with glucose- and serum-free PBS for 40 min. After starvation, the cells were incubated with 2-NBDG (200  µM, Thermo Fisher, Cat#N13195) in PBS for 80 min. Finally, cells were washed once with PBS and cell fluorescence was measured using excitation at 475 nm and emission at 550 nm. Fluorescence positivity was analyzed simultaneously using the Countess 3 FL Automated Cell Counter (Thermo Fisher). Acquired data were statistically compared among control and AdipoRon-treated groups using one-way ANOVA followed by Tukey’s multiple comparisons test.

***Combination treatment using 2-deoxy-D-glucose (2DG)***

MCF7 and MDA-MB-231 cells in 96-well plates were treated with the glycolysis inhibitor 2-deoxy-D-glucose (2DG, 100 and 500 µM) and/or AdipoRon (20 µg/mL) for 24 h, following the previous report (PMID35121743). After treatment, cell viability was measured using the CCK-8 assay. Statistical analysis was performed using one-way ANOVA followed by Tukey’s multiple comparisons test.

***Wound scratch assay***

Cancer cells were seeded in 24-well plates at 1 × 10^5^ cells/well. The medium was replaced with RPMI1640 without FBS to control the cell cycle. The next day, a linear wound was created in each well using a 200-μL tip. The medium was replaced with a control (culture medium with DMSO) or reagent (10, 20, or 40 µg/mL AdipoRon) solution. Images were acquired after 48 h using a Zeiss Cell Discoverer 7 (Zeiss, Gottingen, Germany), and the distance between the wound edges was measured using Cell Discoverer 7. The wound healing rate was calculated by dividing the distance at 48 h by that at 0 h. Data are shown as mean ± SD from at least two independent experiments with duplicate or triplicate samples.

***Combination treatment of AdipoRon and conventional therapeutic reagents***

Human breast cancer cell lines were treated with DMSO (Sigma-Aldrich, Cat#D5879) as control, AdipoRon (20 µg/mL), 4-hydroxytamoxifen (TAM, Sigma-Aldrich, Cat#H6278) at 5 or 10 µM, paclitaxel (PTX, Cayman chemical, Ann Arbor, MI, USA. Cat#10461) at 5 or 20 nM, or doxorubicin (DOX, Cayman chemical, Cat#15007) at 0.1 or 2 µM, alone or in combination. After 72 hours, cell proliferation was measured using CCK-8, and cell viability was calculated relative to untreated control cells. Statistical analysis was performed usin one-way ANOVA followed by an unpaired two-tailed Welch’s t-test.

***Combination treatment in cells with manipulated AdipoR1 expression.***

Human breast cancer cell lines with manipulated AdipoR1 expression were treated with 20 μg/mL AdipoRon, TAM at 5 or 10 µM, or DOX at 1 µM, alone or in combination. After 72 hours, cell proliferation was measured using CCK-8, and cell viability was calculated relative to untreated control cells. Statistical analysis was performed using one-way ANOVA followed by an unpaired two-tailed Welch’s t-test.

***Immunohistochemistry (IHC) using tissue microarray cores***

Tissue microarrays (TMAs) prepared from anonymized paraffin-embedded tissue blocks of breast cancer from included patients were used for immunostaining. In brief, TMA blocks were sectioned at 5-μm thickness. After antigen retrieval using a commercially available solution (415211; Nichirei Biosciences, Tokyo, Japan) or proteinase (P8038; Sigma-Aldrich), the sections were incubated with antibodies against AdipoR1 (ab70362, 1:200, rabbit polyclonal IgG Ab; Abcam) at room temperature (20–25°C) for 1 h. The concentrated antibodies were diluted in SignalStain Antibody Diluent (#8112, Cell Signaling Technology, Danvers, MA, USA). After washing with PBS, the sections were incubated with ready-to-use biotinylated species-specific secondary antibodies (724132, 724142; Nichirei Biosciences), washed, and incubated with 0.5 mg/mL 3,3′-diaminobenzidine (725191; Nichirei Biosciences) for visualization. Non-specific mouse or rabbit IgG was used as a negative control.

***Apoptosis assay***

Apoptosis was assessed using a cleaved caspase 3/7 detection kit (Invitrogen). After exposing cells to the kit reagent for 30 min, green fluorescence-stained cells were counted using a Countess 3 FL automated cell counter (Thermo Fisher Scientific). The assay was repeated three times independently, and average values were calculated. The data are shown as mean ± SD from at least two independent experiments with duplicate or triplicate samples.

***Ferroptosis assay***

Ferroptosis was assessed using a Liperfluo ferroptosis detection kit (Cat# L248; Dojindo). Trypsinized cells were incubated with Liperfluo in an incubator for 1 h. Then, the cells were washed with Hank’s balanced salt solution (Cat# 14025092; Thermo Fisher Scientific), and green fluorescence-stained cells were counted using the Countess 3 FL automated cell counter. The assay was repeated three times independently, and average values were calculated. Data are shown as mean ± SD from at least two independent experiments with duplicate or triplicate samples.

***In vivo experiments***

All animal experiments conformed to the ARRIVE guidelines and were approved by the Kanagawa Cancer Center Animal Experimentation Committee (approval number 01-07). Ten-week-old female C57BL6 (Jackson Laboratory Japan, Yokohama, Japan, RRID:IMSR_JAX:000664) mice were subcutaneously implanted with 1 × 10^6^ EO771 cells on the left and right flanks. Mice were randomly assigned to groups to ensure that littermates and body weight were balanced across groups. Outcome assessments were performed by a separate investigator who was not involved in the experimental procedures and was blinded to the group assignments. Mice in the control group received 0.4% cellulose, whereas those in the treatment group received 50 mg/kg AdipoRon (Adipogen) i.p. or i.g. five times a week for two weeks. Tumor diameters were measured twice a week during the treatment. Two weeks after treatment, the mice were sacrificed, and the tumors were excised and weighed. The experiment was performed three times with *n* = 3 mice in each of the control, i.p, and i.g groups. The exclusion criteria were predefined as follows: animals were excluded if their tumor long axis exceeded 20 mm or if they experienced more than 20% body weight loss. Tumors and organs collected from the animals were fixed in 10% neutral buffered formalin for 24 h, embedded in paraffin, and prepared as formalin-fixed paraffin-embedded (FFPE) samples for subsequent histopathological analysis. The FFPE blocks were sectioned at 4 µm, and the sections were stained with hematoxylin and eosin (H&E). Additional sections were used for immunohistochemical analysis. After antigen retrieval using a commercially available solution (415211; Nichirei Biosciences) or proteinase (P8038; Sigma-Aldrich), the sections were incubated with antibodies against Erythropoietin (EPO) (66975-1-Ig, 1:200, mouse monoclonal IgG Ab; Proteintech Group, Rosemont, IL, USA) and HIF2α (AF2997, 1:200, goat polyclonal IgG Ab; R&D Systems, Inc., Minneapolis, MN, USA) at room temperature (20°C–25°C) for 1 h. The concentrated antibodies were diluted in SignalStain Antibody Diluent (#8112, Cell Signaling Technology). After washing with PBS, the sections were incubated with ready-to-use biotinylated species-specific secondary antibodies (724132, 724142; Nichirei Biosciences), washed, and incubated with 0.5 mg/mL 3,3′-diaminobenzidine (725191; Nichirei Biosciences) for visualization. Non-specific mouse or rabbit IgG was used as a negative control. Immunohistochemical staining was evaluated using the H-score method, calculated as the product of the percentage of positive cells (0–100) and the staining intensity (0–3), yielding a total score of 0-300.

***RNA-sequencing (RNA-seq) analysis***

Human breast cancer cells (MCF7, T47D, MDA-MB-231, and MDA-MB-468) were exposed to AdipoRon at 10 µg/mL or 40 µg/mL or to dimethyl sulfoxide (DMSO; Cat# 043-07216; Fujifilm Wako) as a control. After 24 h of exposure, RNA was extracted from all cells using an RNeasy Mini kit (Qiagen). RNA quality was assessed using a Nanodrop (Thermo Fischer). Sequencing libraries were prepared from qualified total RNA using the SMARTer Stranded Total RNA Sample Prep Kit (635005; Takara Bio) and sequenced using Illumina NextSeq500 (Illumina), generating 236-bp reads. RNA-seq was outsourced to Takara Bio. Differentially expressed genes were identified based on *P* < 0.05 (*t*-test) and log2(fold change) > 1 and functionally annotated using Gene Ontology and Kyoto Encyclopedia of Genes and Genomes pathway enrichment analysis (KEGG). Ingenuity Pathway Analysis (IPA, Qiagen, Hilden, Germany) was conducted using our original datasets. The sequence reads produced in this study are deposited at Gene Expression Omnibus (GEO) under accession No. GSE290491.

***In silico analysis using public data***

*In silico* analysis was performed using data from cBioPortal, the Cancer Cell Line Encyclopedia (CCLE; https://sites.broadinstitute.org/ccle/), and the Human Protein Atlas. The mRNA expression and gene amplification/deletion of 37 adipokines and adipokine receptors was analyzed using cBioPortal, based on RNA-seq data of tumor tissues from cancers originating in various organs. AdipoR1 expression status in the various cancer types was analyzed using the search tool of the Human Protein Atlas. ER, PgR, HER2, and AdipoR1 mRNA expression in breast cancer cell lines was analyzed using the CCLE website.

*In silico* analysis of AdipoRon sensitivity in cancer cell lines using public databases was conducted. To this end, a correlation analysis between cell proliferation and AdipoR1 mRNA expression was performed using cell proliferation data following treatment with 2.5 µM (0.87 µg/mL) AdipoRon and AdipoR1 mRNA expression data for 527 cell lines in the DepMap portal (DepMap 24Q2 Public release). Correlation analysis between AdipoRon sensitivity and AdipoR1 mRNA expression data in thirteen individual breast cancer cell lines was also performed using data from DepMap and the Human Protein Atlas.

***Lentiviral transfection***

Stable gene knockdown or expression was achieved using the ViraPower Lentiviral expression system (Invitrogen) according to the manufacturer’s protocol. Gene manipulation from polyclonal stable cell lines was checked using western blotting. Lentiviral shRNA constructs targeting *ADIPOR1* were from Sigma-Aldrich. Stable *ADIPOR1* overexpression was achieved by cloning the open reading frame of *ADIPOR1* downstream of the CMV promoter in the pLOC lentiviral vector (Dharmacon).

# *Statistical analysis*

Statistical tests were chosen based on the experimental design and data distribution. Normality of each dataset was assessed using the default D’Agostino–Pearson omnibus normality test in GraphPad Prism 7.0 software (SCR_002798, GraphPad Software, La Jolla, CA, USA) prior to applying parametric tests. Measures of variation (mean ± standard deviation or standard error of the mean) are reported for all data. Only datasets meeting the assumptions of normality were analyzed using parametric tests such as t-tests or ANOVA, while non-parametric tests were applied when normality assumptions were not satisfied. Variances between groups were confirmed to be comparable prior to analysis. Data are reported as mean ± SD and were analyzed using GraphPad Prism 7.0 software. Means of two groups were compared using Student’s unpaired *t*-test. For multiple group comparisons, an ordinary ANOVA was employed, followed by the Tukey–Kramer or Dunnett's multiple comparison tests. Statistical significance was set to *P* < 0.05. Pearson’s chi-squared test was used to determine correlations between clinical parameters and overall survival. In Ingenuity Pathway Analysis (SCR_008653, Qiagen), canonical pathway activation was assessed based on the z-score, with a positive value indicating pathway activation and a negative value indicating pathway inhibition. Kaplan–Meier plots were generated using the Kaplan-Meier plotter ^66^. Simple linear regression analysis was performed to assess the relationship between AdipoR1 mRNA expression and the effect of AdipoRon on cell growth.

**Ethical approval**

This study was approved by the Kanagawa Cancer Center Ethics Committee (approval number: 2020 EKI-37) and conducted in accordance with the study protocol approved by the center. Comprehensive written informed consent was obtained from all patients included in the study. All animal experiments conformed to the ARRIVE guidelines and were approved by the Kanagawa Cancer Center Animal Experimentation Committee (approval number 01-07). All methods, including study design, sample size, inclusion and exclusion criteria, outcome measures, method of euthanasia, timing of tissue collection after euthanasia, and statistical methods, were performed according to relevant guidelines and regulations.

**Conflict of interest:**

S. Sato received research funding from Nikon Corporation and Takeda Pharmaceutical Company. Y. Miyagi received research funding from Tosoh Corporation and Shin Nippon Biomedical Laboratories, Ltd. T. Yamashita received research grants and/or honoraria for lectures from Chugai, Eisai, Novartis Pharma, Taiho, Nippon Kayaku, AstraZeneca, Kyowa Kirin, Pfizer Japan, Eli Lilly, and Daiichi Sankyo. T. Yamanaka received honoraria for lectures from Chugai, Eisai, Novartis Pharma, AstraZeneca, Kyowa Kirin, Pfizer Japan, Eli Lilly, and Daiichi Sankyo. All other authors declare no conflicts of interest.

**Availability of data and materials:**

The RNA-seq data and original western blot images were deposited at GEO under GEO:233867 and Mendeley at 10.17632/hrw8kxcsxp.1, respectively, and are publicly available as of the date of publication. Microscopy data reported in this paper will be shared by the lead contact upon request. Any additional information required to reanalyze the data reported in this paper is available from the lead contact upon request.

**Acknowledgements:**

We thank Dr. Yutaka Kondo (Nagoya University), Dr. Naohiko Koshikawa (Institute of Science Tokyo), and Dr. Takuro Nakamura (Tokyo Medical University) for providing cogent and meaningful suggestions; Ms. Yoshihara and Ms. Ohrui for preparing the specimens; Ms. Nakagawa for support in the *in vitro* experiments; Ms. Kasashima for cleaning the RNA-seq data and processing the GSEA data; Dr. Daisuke Hoshino for providing us with 4T1-Luc cells; and Dr. Emi Yoshioka, Dr. Kae Kawachi, Dr. Kota Washimi, Dr. Yoichiro Okubo, and Dr. Tomoyuki Yokose for preparing pathological breast cancer specimens. This work was supported by the Japan Society for the Promotion of Science (JSPS) Grants-in-Aid for Scientific Research (KAKENHI; grant No. 23K08708 to Shinya Sato).

# Author’s contributions

Sato S contributed to the study concept, TCGA data analysis, RNA-seq data analysis, extraction of candidate genes from the data, cell and animal experiments, histopathological analysis of specimens, and manuscript writing. Komori Y and Ishida M performed cell and animal experiments. Nakamura Y performed immunohistochemistry. Miyagi Y, Yamashita T, and Yamanaka T provided advisory guidance on the concept, and Yamashita T and Yamanaka T prepared the breast cancer tissues.

**Figure legends of supplementary figures**

**Supplementary Figure S1**

**Additional gene expression analysis of breast cancer tissues and cell lines using public databases.**

A-D: Gene expression analysis of adipokines and adipokine receptors in breast cancer of several datasets. E: Estrogen receptor, Progesterone receptor, HER2, and AdipoR1 mRNA expression of breast cancer cell lines in Cancer Cell Line Encyclopedia (CCLE). F: A chemical structural formula of the AdipoRon. G: AdipoR1 mRNA expression across cancer types in the Human Protein Atlas.

**Supplementary Figure S2**

**Effects of AdipoRon on mitochondrial stress and glucose metabolism.**

**A,** Protein expression of oxidative phosphorylation (OXPHOS)-related proteins, Tom20, and UCP2 in breast cancer cell lines 48 h after AdipoRon exposure (10 or 40 µg/mL). C, control (DMSO); A10, 10 µg/mL AdipoRon; A40, 40 µg/mL AdipoRon.

**B, Top:** Snapshots of cells positive for 2-(N-(7-nitrobenz-2-oxa-1,3-diazol-4-yl)amino)-2-deoxyglucose (2-NBDG) detected by the Countess 3 FL. Quantification of 2-NBDG-positive cells in MCF7 and MDA-MB-231 cells with or without AdipoRon treatment (25 and 50 µM).
**Bottom:** Relative cell viability following treatment with the glycolysis inhibitor 2-deoxy-D-glucose (2DG, 100 and 500 µM) and/or AdipoRon (20 and 40 µg/mL) in MCF7 and MDA-MB-231 cells. ***P < 0.001; one-way ANOVA followed by Tukey’s multiple comparison test.

**C, Top:** Representative immunohistochemical images of Tom20- and UCP2-stained breast cancer tissues with or without AdipoRon treatment.
**Bottom:** H-scores of Tom20 and UCP2 in breast cancer tissues from control and AdipoRon-treated mice. n.s., not significant; unpaired two-tailed Welch’s t-test.

**Supplementary Figure S3**

**Correlation between cell growth effects of AdipoRon and AdipoR1 mRNA expression in cell lines from public databases.**

A. Correlation analysis of the inhibitory (left) and promoting (right) effects of AdipoRon (2.5 µM) on cell growth and their association with AdipoR1 mRNA expression in the 527 cancer cell lines.

B. Correlation analysis of the inhibitory (left) and promoting (right) effects of AdipoRon (2.5 µM) on cell growth and their association with AdipoR1 mRNA expression in 13 breast cancer cell lines. Simple linear regression was used to assess correlations.

**Supplementary Figure S4**

**Combination treatment in cells with manipulated AdipoR1 expression.**

CCK-8 assay of human breast cancer cell lines stably expressing shRNA targeting AdipoR1 (sh) or overexpressing AdipoR1 (OE) after AdipoRon treatment. All cell lines were treated with 20 µg/mL AdipoRon. MCF7 cells were treated with 5 µM 4-Hydroxytamoxifen (TAM), T47D cells with 10 µM TAM, and MDA-MB-231 cells with 1 µM Doxorubicin hydrochloride (DOX). After 72 hours, cell proliferation was measured using CCK-8, and cell viability was assessed relative to untreated control cells. ***P < 0.001; one-way ANOVA followed by Dunnett’s multiple comparison tests.

**Supplementary Figure S5**

**Ferroptosis assay, additional gene set enrichment analysis, and specific proteins and mRNAs regulated by AdipoRon.**

A: Ferroptosis assay of breast cancer cell lines after the indicated treatments. Lipid peroxide was detected after 24 h of AdipoRon (10 or 40 µg/mL) or DMSO exposure. Left panel: representative image of MCF7 cells treated or not with AdipoRon. Right panel: Lipid peroxide positivity after AdipoRon treatment in breast cancer cell lines. ****p* < 0.001; one-way ANOVA followed by Dunnett’s multiple comparison tests. B: Gene set enrichment analysis related to lipid synthesis, insulin resistance, and TN-specific regulated pathways of breast cancer cell lines with or without AdipoRon treatment. C: Count data of RN-Seq analysis of Estrogen receptor, BRCA1, and BRCA2 in breast cancer cell lines with or without AdipoRon treatment. D: Top: Protein expression of TROP2 in MCF7 with or without AdipoRon treatment (20 or 40 µg/mL). Bottom: TROP2 mRNA counts from RNA-Seq results. ****p* < 0.001; unpaired two-tailed Welch’s t-test.

**Supplementary Figure S6**

Effect of AdipoRon treatment on body weight, liver histology, and hepatic expression of aneima-related proteins in tumor-bearing mice. A, top panel: The body weight of mice transplanted with EO771 cells, with or without AdipoRon treatment (n = 9 per group). One-way ANOVA followed by Dunnett’s multiple comparison tests was performed. Bottom panel: Representative images of H&E-stained livers of control (0.4% cellurose) and AdipoRon-treated mice. Scale bar = 100 µm. B, top panel: Representative images of erythropoietin (EPO) and HIF2α immunohistochemistry (IHC) in the livers of control and AdipoRon-treated mice. Scale bar = 100 µm. Bottom panel: H-scores of EPO and HIF2α in the livers of control and AdipoRon-treated mice. n.s., not significant; unpaired two-tailed Welch’s t-test.
