# Supplementary figures and images for: AdipoR1–AMPK axis suppresses breast cancer across molecular subtypes via multimodal cell death pathways, including ferroptosis and apoptosis
Source: Cell Death Dis. 2026 Mar 26;17(1):384. doi: 10.1038/s41419-026-08583-7 (PMC13049035; doi:10.1038/s41419-026-08583-7)

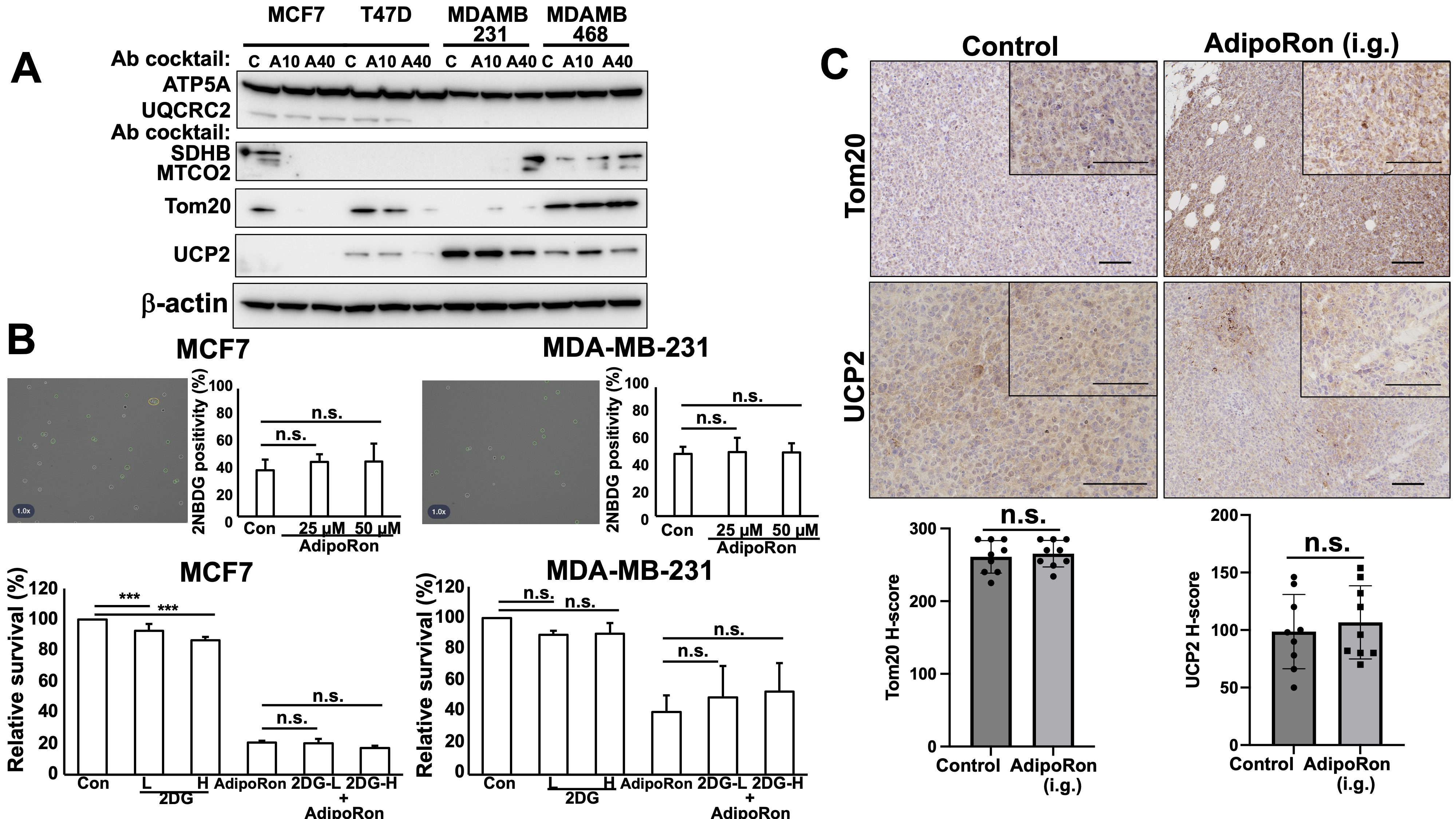

Supplement: Supplementary file 3 — Supplementary Figure S2 [file 41419_2026_8583_MOESM3_ESM.tif]

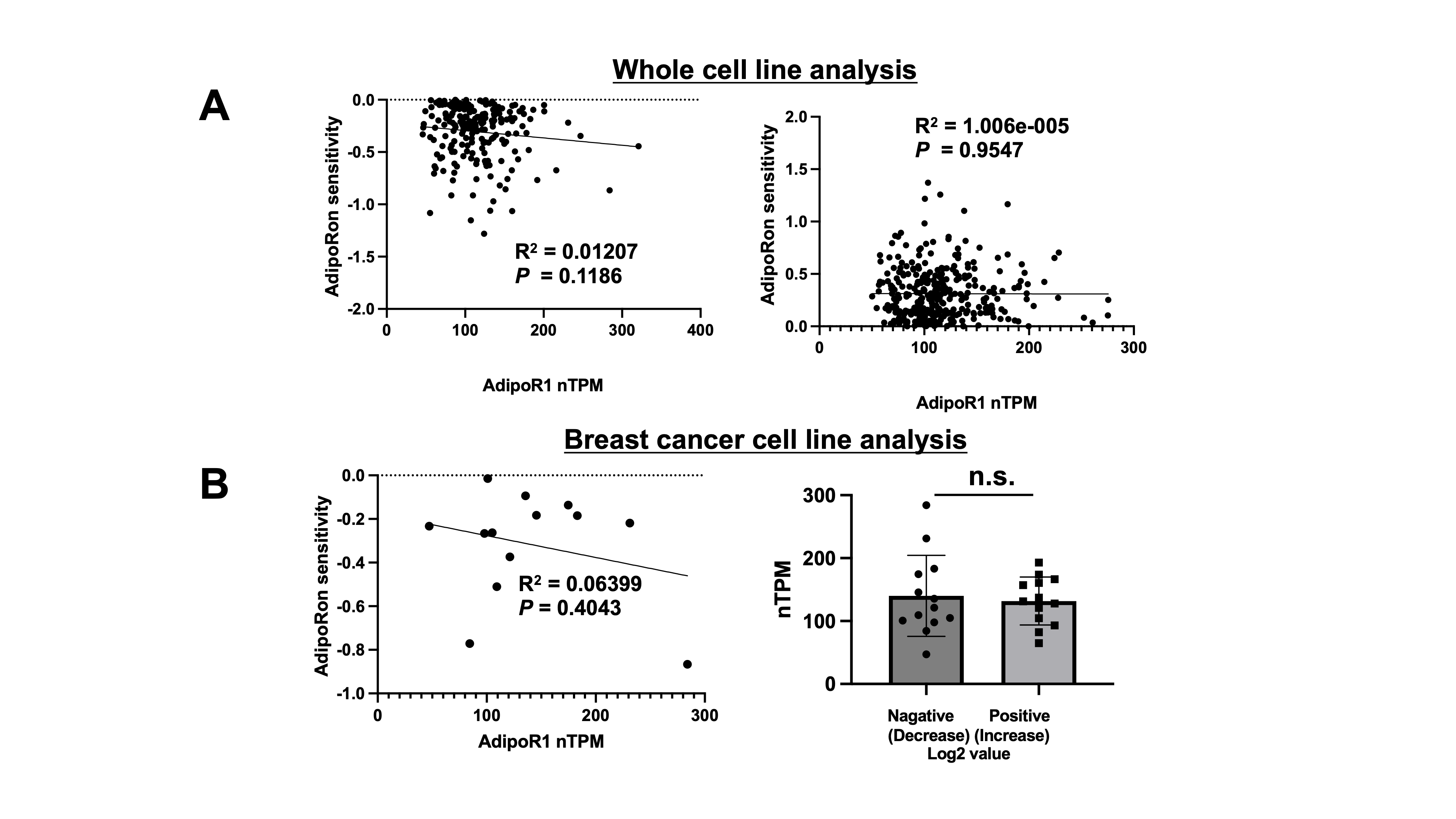

Supplement: Supplementary file 4 — Supplementary Figure S3 [file 41419_2026_8583_MOESM4_ESM.tif]

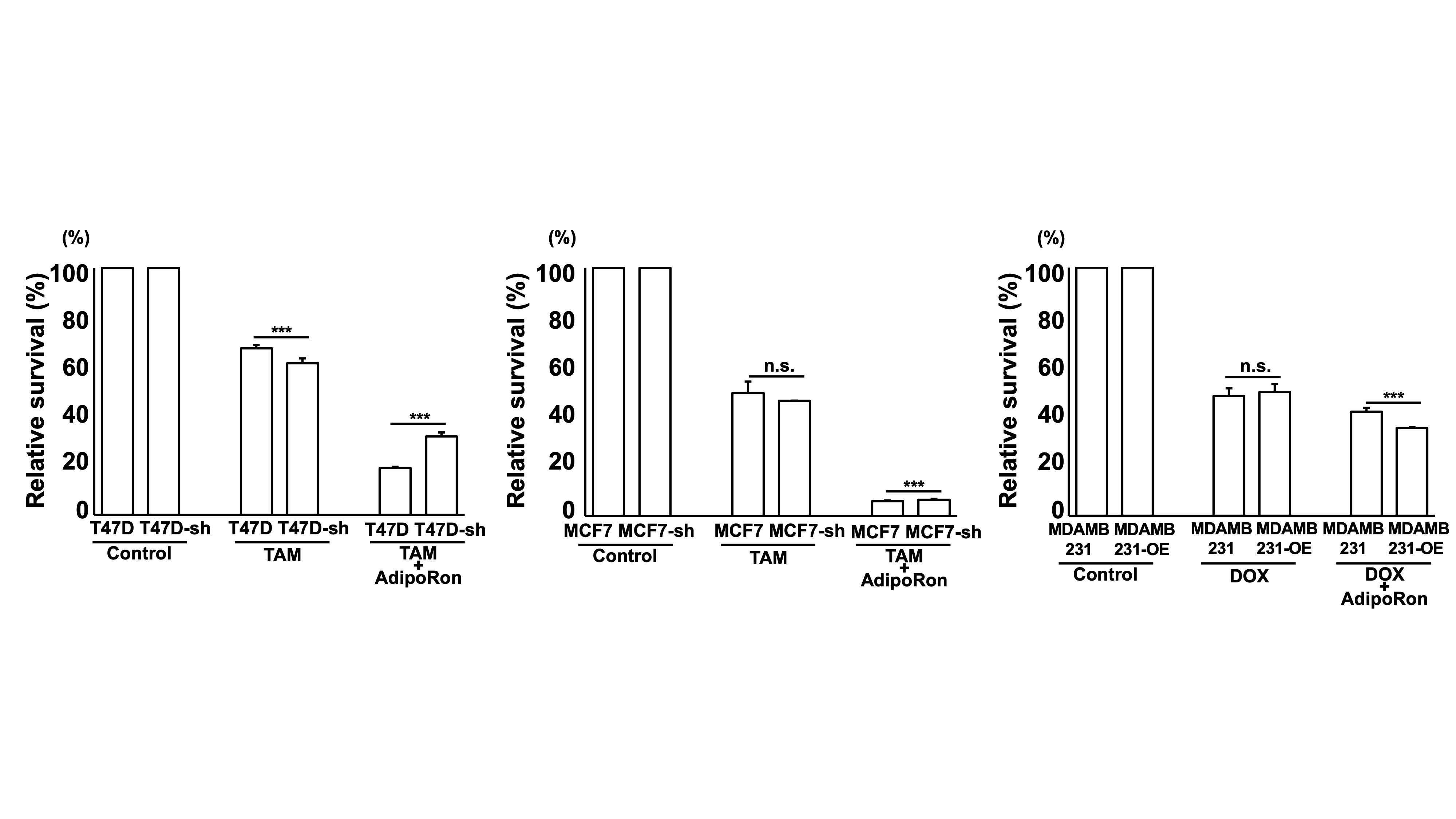

Supplement: Supplementary file 5 — Supplementary Figure S4 [file 41419_2026_8583_MOESM5_ESM.tif]

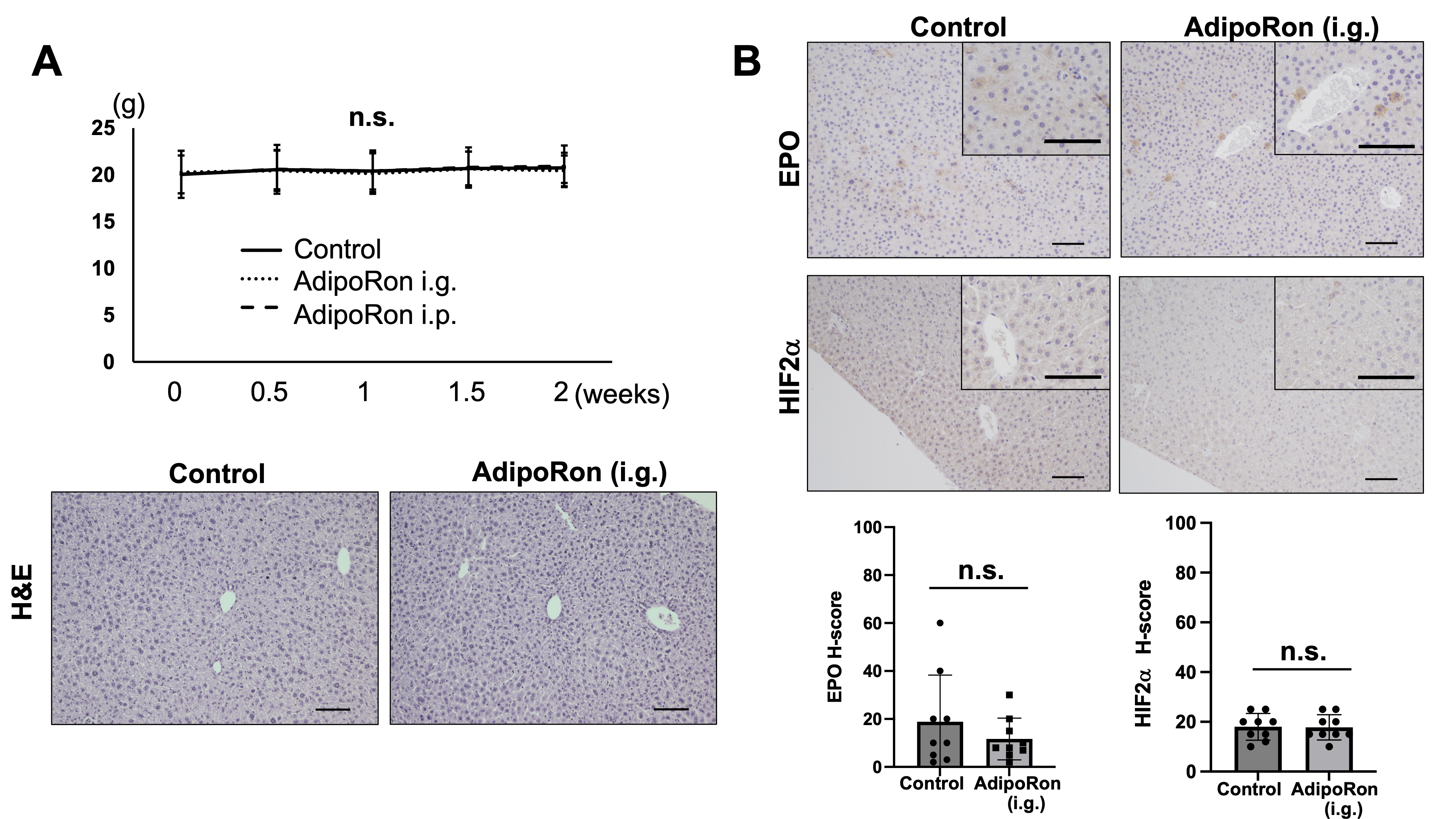

Supplement: Supplementary file 7 — Supplementary Figure S6 [file 41419_2026_8583_MOESM7_ESM.tif]
