# Supplementary material for: AdipoR1–AMPK axis suppresses breast cancer across molecular subtypes via multimodal cell death pathways, including ferroptosis and apoptosis
Source: Cell Death Dis. 2026 Mar 26;17(1):384. doi: 10.1038/s41419-026-08583-7 (PMC13049035; doi:10.1038/s41419-026-08583-7)

Figure 2A, 3D

western blot 12-26-2024
P362-No.155
1st： Adiponectin Receptor 1 Rabbit IgG Affinity Purify抗体(18993)MBL(100倍) 2024/12/24-2024/12/26 4℃ o/n
2nd：rabbit(5,000倍) 2024/12/26 RT 1h
ChemiDoc：Immunostar

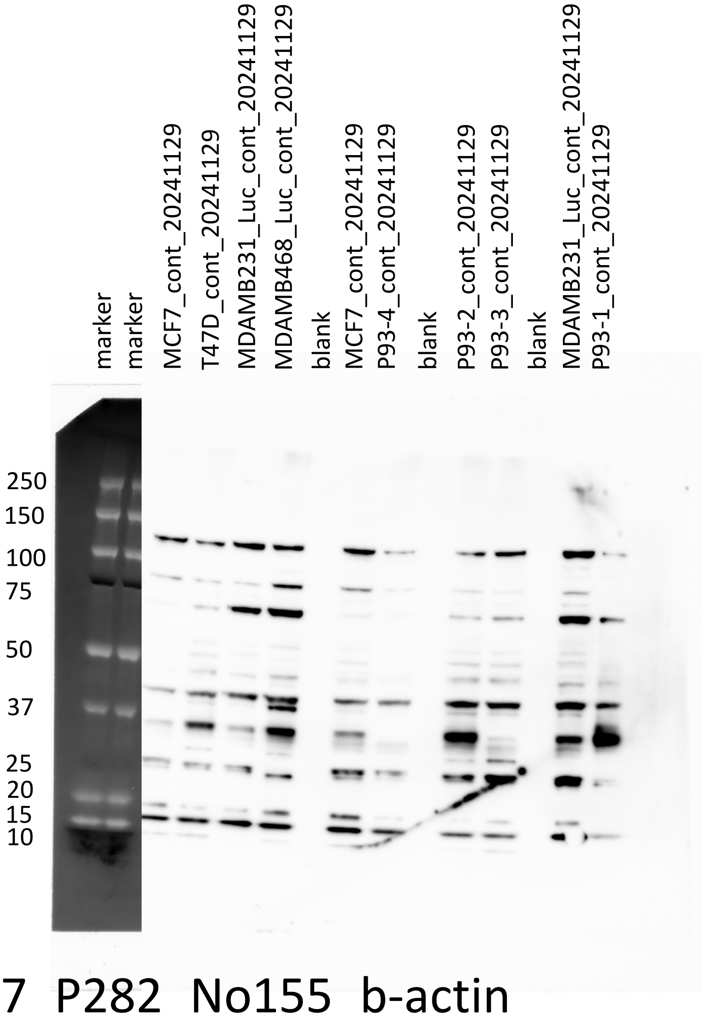

2025/1/7_P282_No155_b-actin

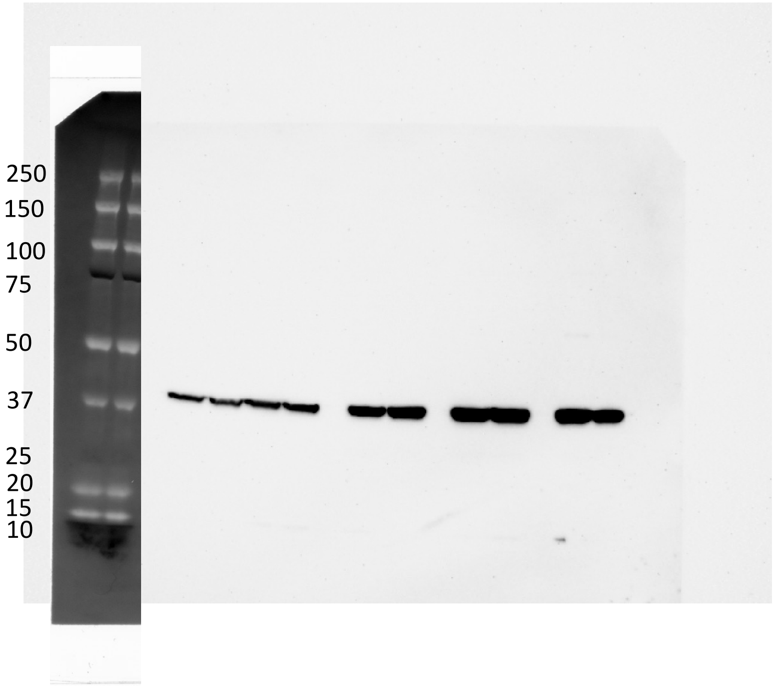

Figure 2C

2024/6/25_P282_No131_p-AMPK_CST2535

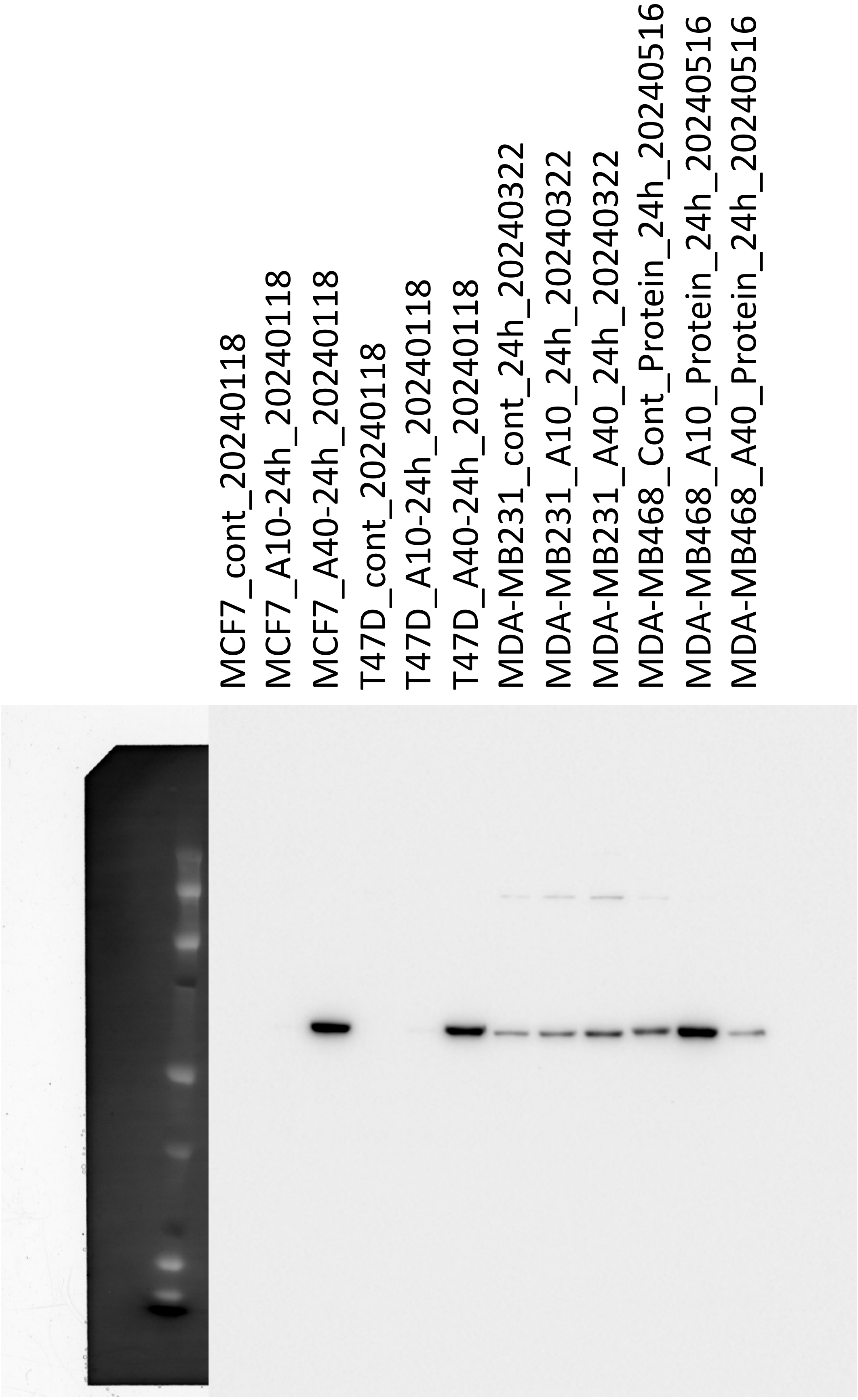

Figure 2C

2024/6/26 _P282_No131_AMPK_CST5831

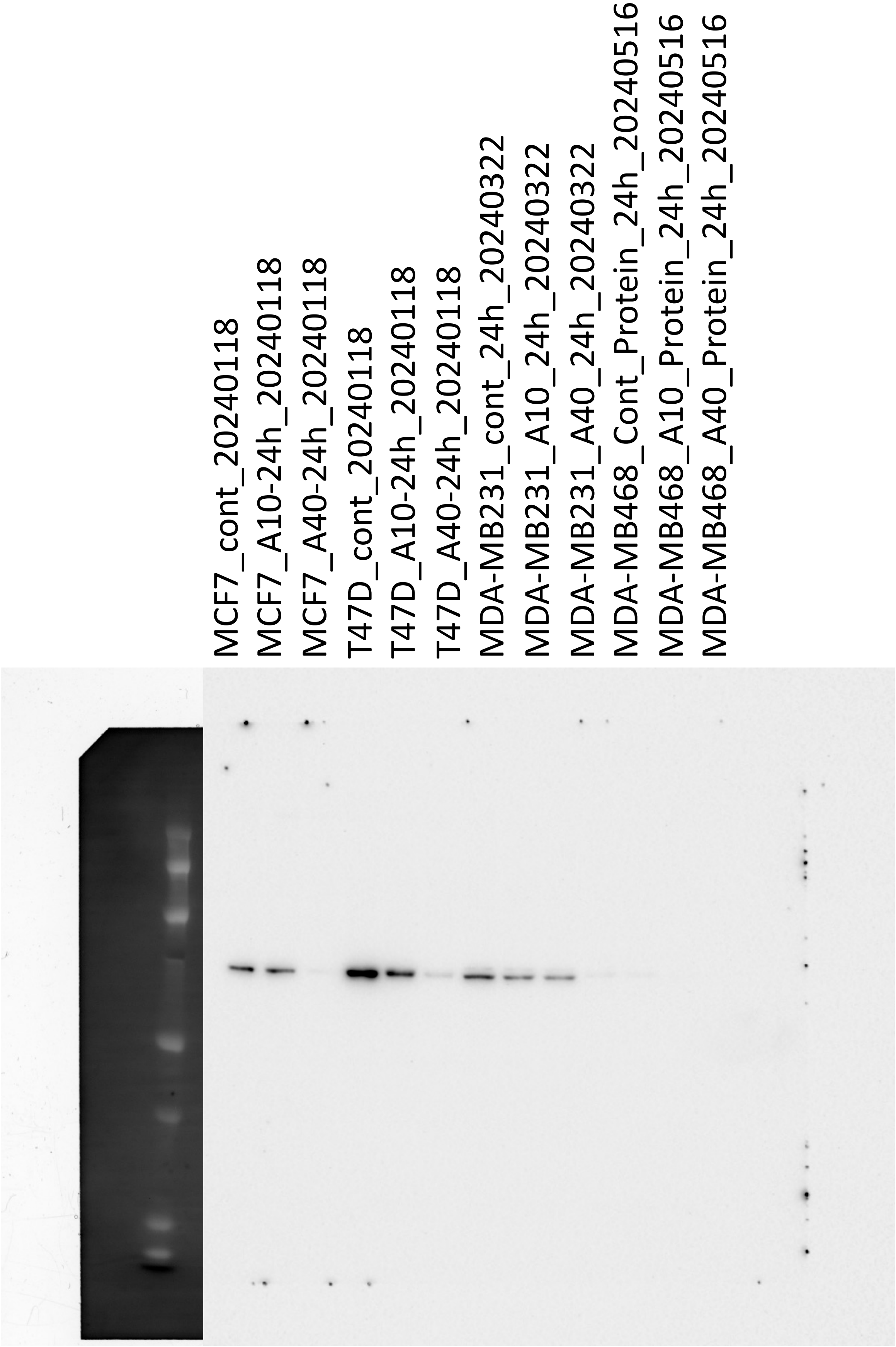

Figure 2C

2024/7/5 _P282_No131_b-actin

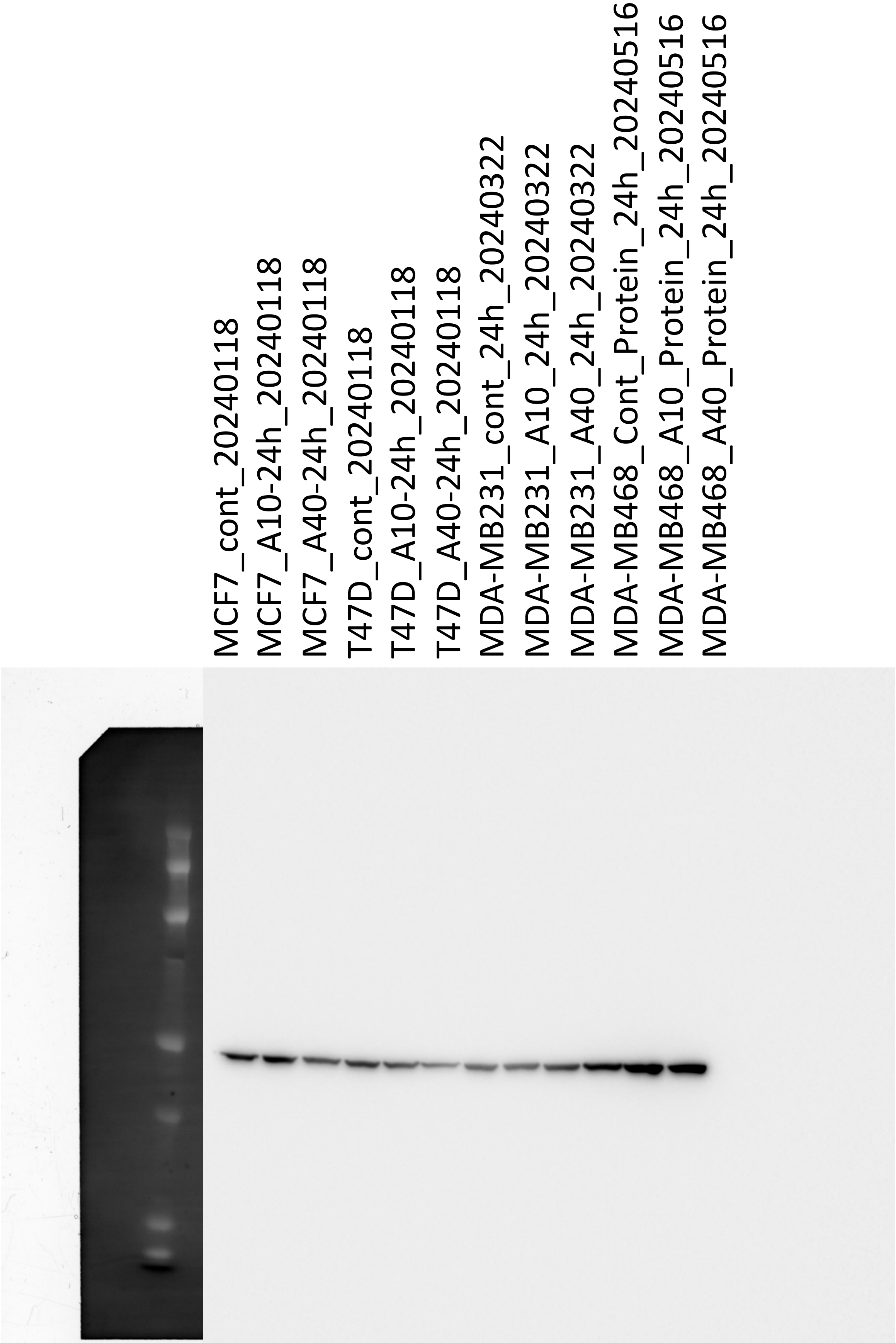

Figure 2C

2024/5/23　No127　p-AMPK　CST2535

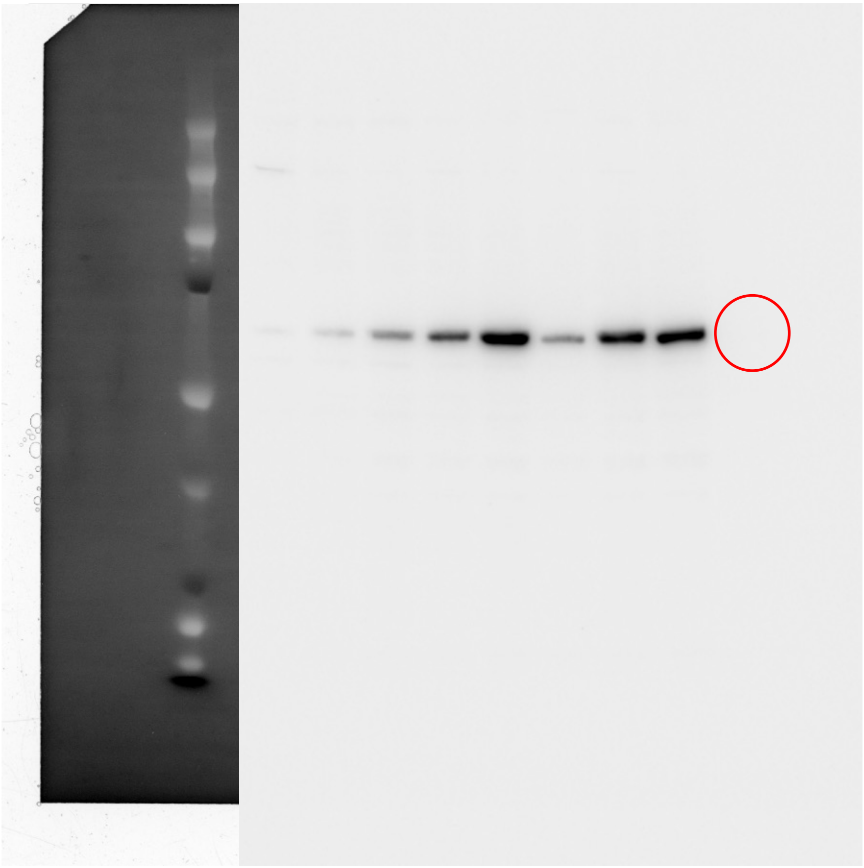

Figure 2C

2024/5/28 No127 AMPK CST5831

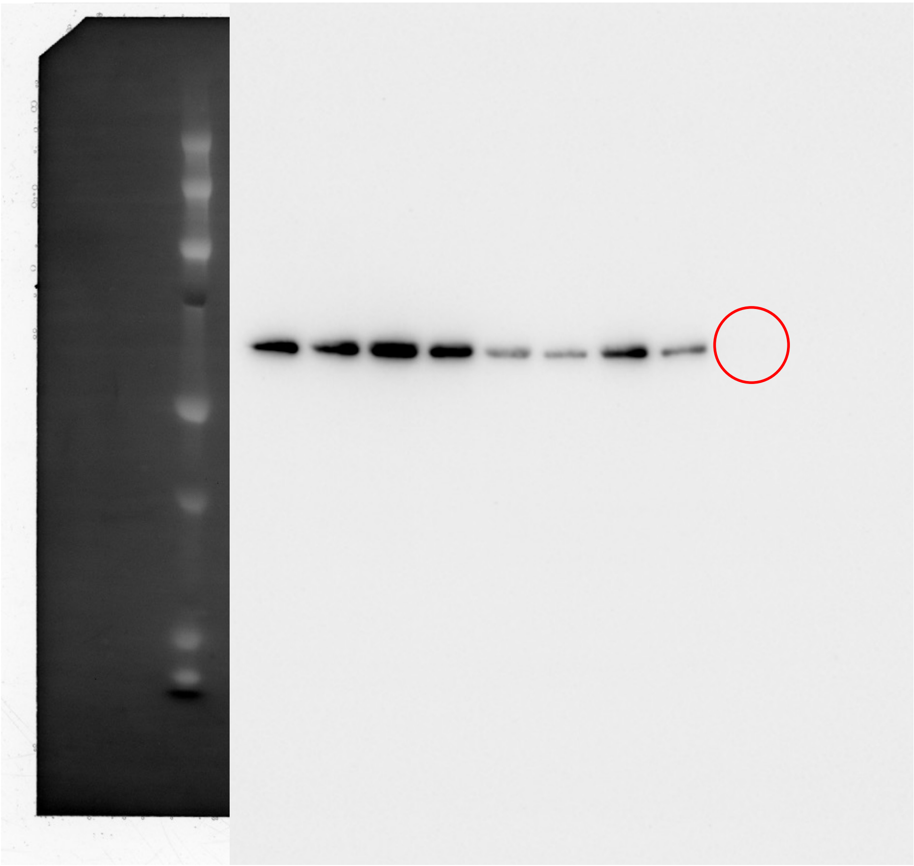

Figure 2C

2024/6/10 No127 β-actin CST4970

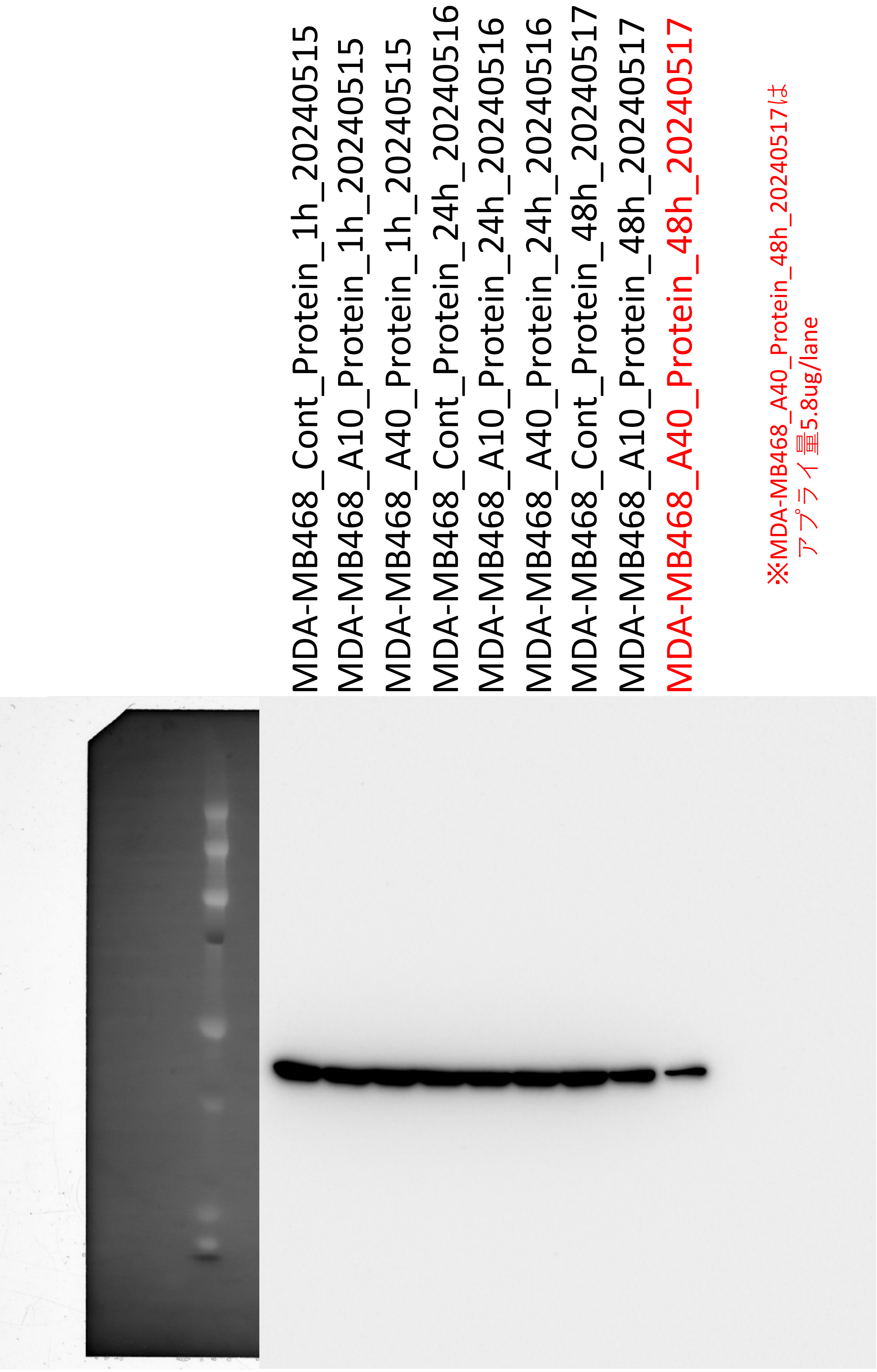

Figure 2C

20240625_pAMPK

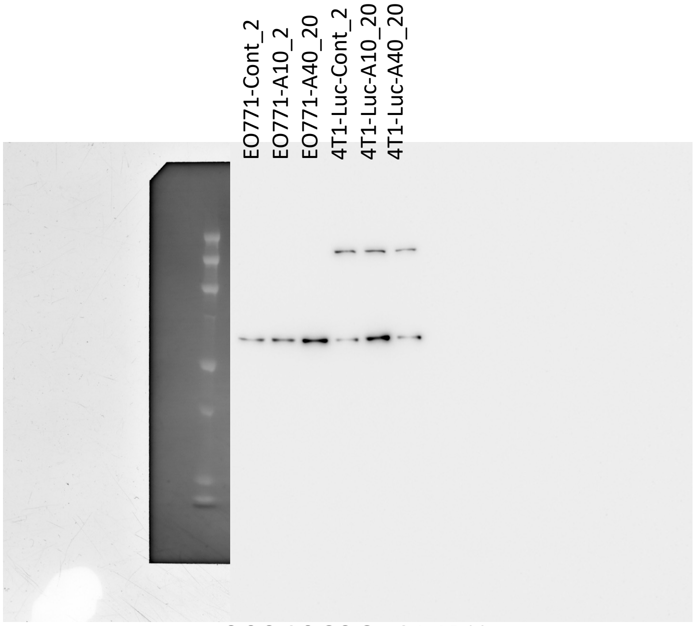

20240626_AMPK

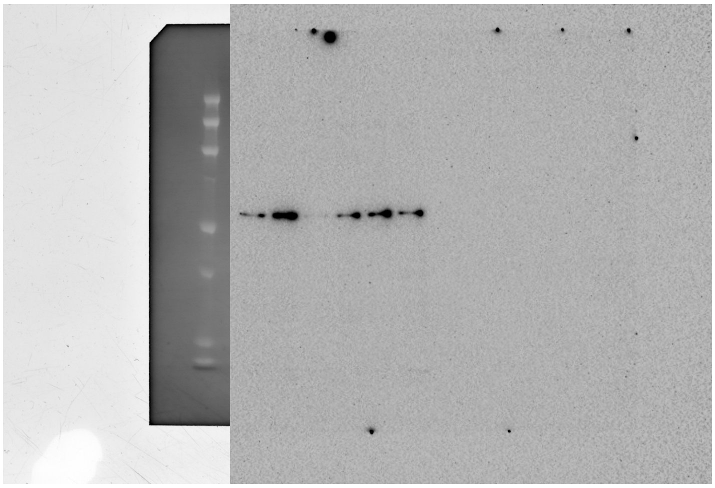

202406702_b-actin

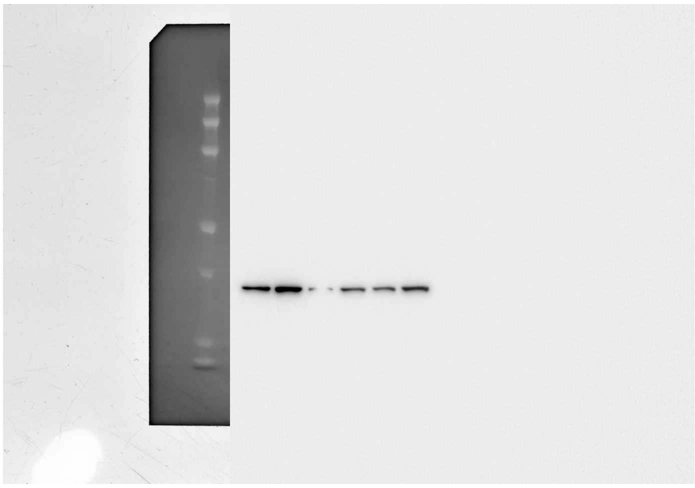

Figure 2C

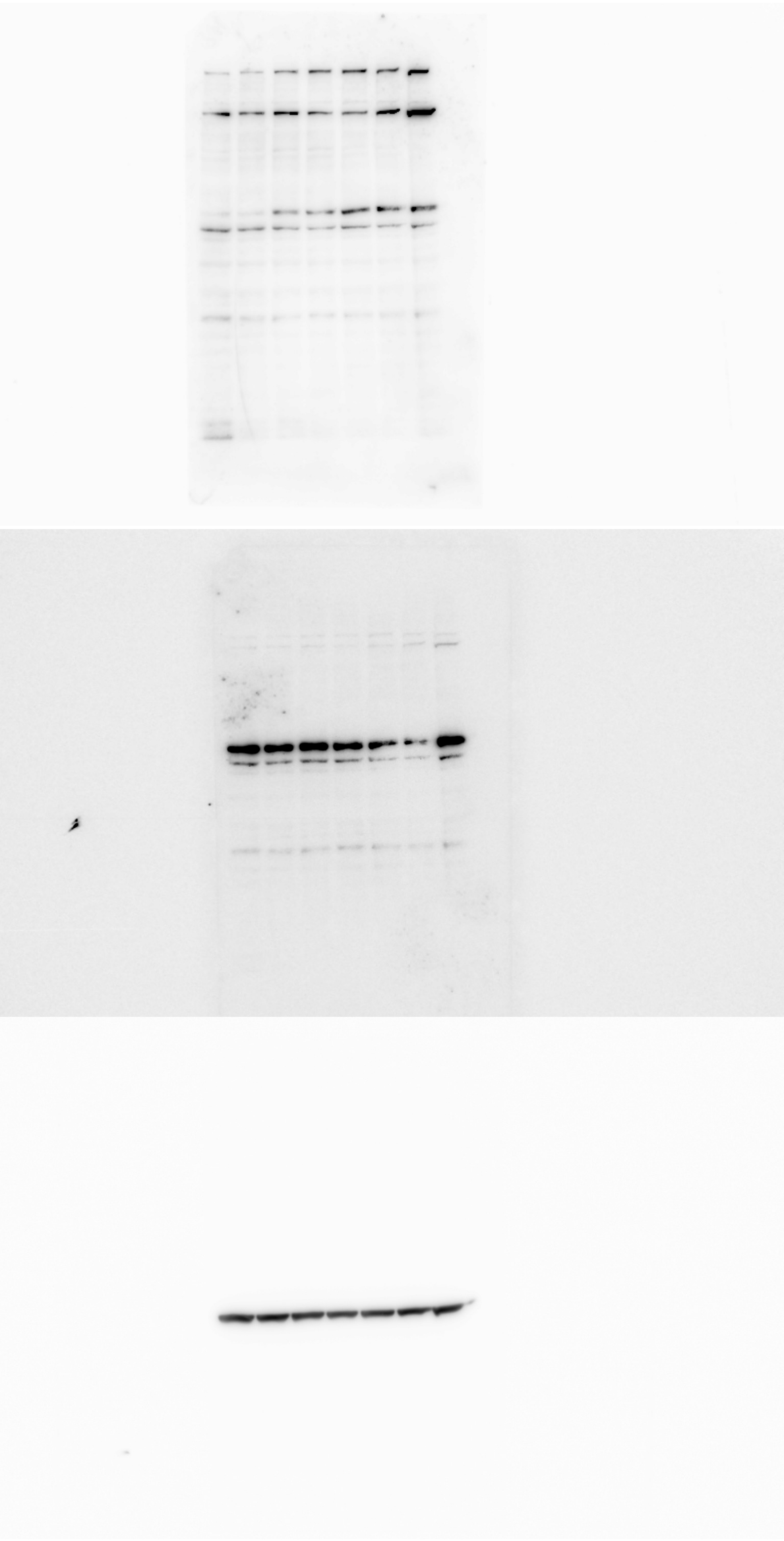
Chronological exposure of AdipoRON
4T1-Luc (A40) No63
C
0
5
10
15
30
40 (min)
2022-7-4AMPK
2022-7-12-AMPK
β-actin

Figure 4C

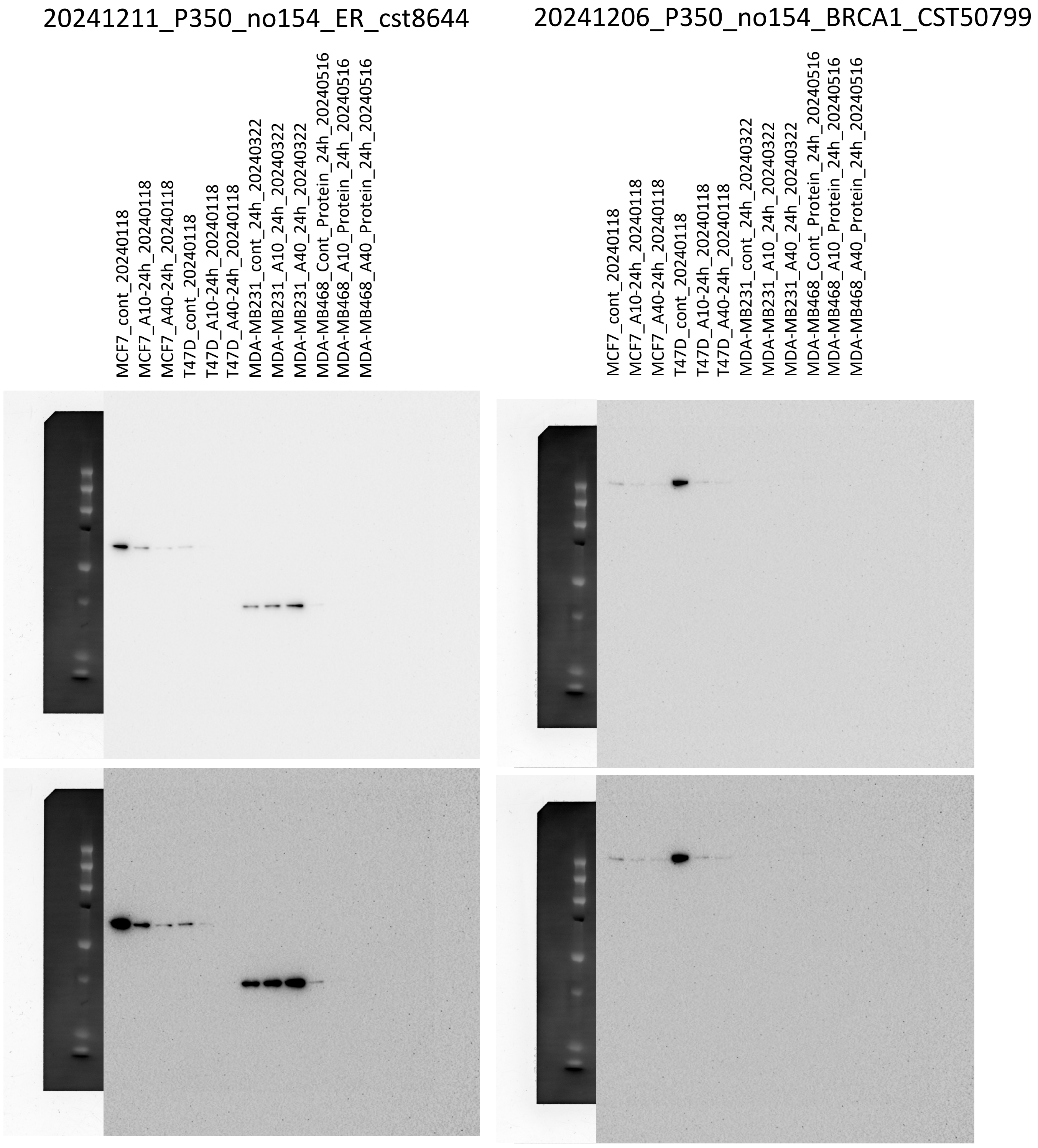

MCF7_cont_20240118
MCF7_A10-24h_20240118
MCF7_A40-24h_20240118
T47D_cont_20240118
T47D_A10-24h_20240118
T47D_A40-24h_20240118
MDA-MB231_cont_24h_20240322
MDA-MB231_A10_24h_20240322
MDA-MB231_A40_24h_20240322
MDA-MB468_Cont_Protein_24h_20240516
MDA-MB468_A10_Protein_24h_20240516
MDA-MB468_A40_Protein_24h_20240516

Figure 4C

20241120_P350_no154_BRCA2_CST10741

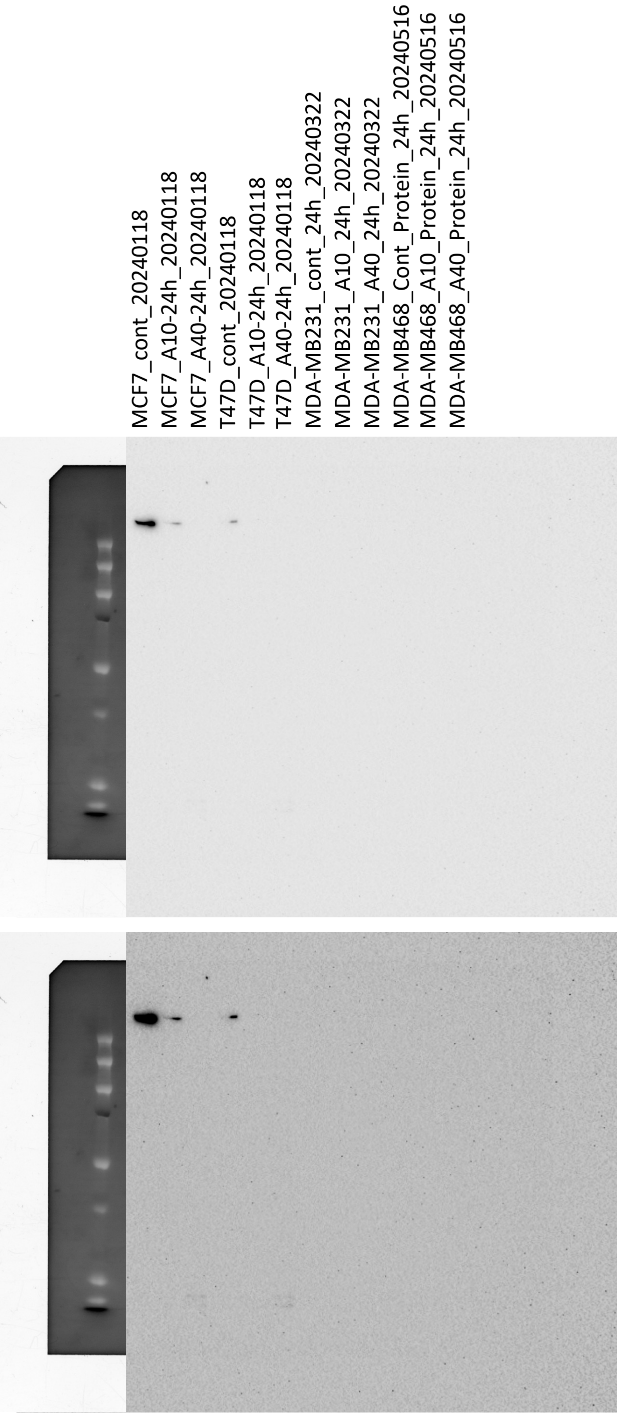

20241219_cell line_b-actin_cst4970

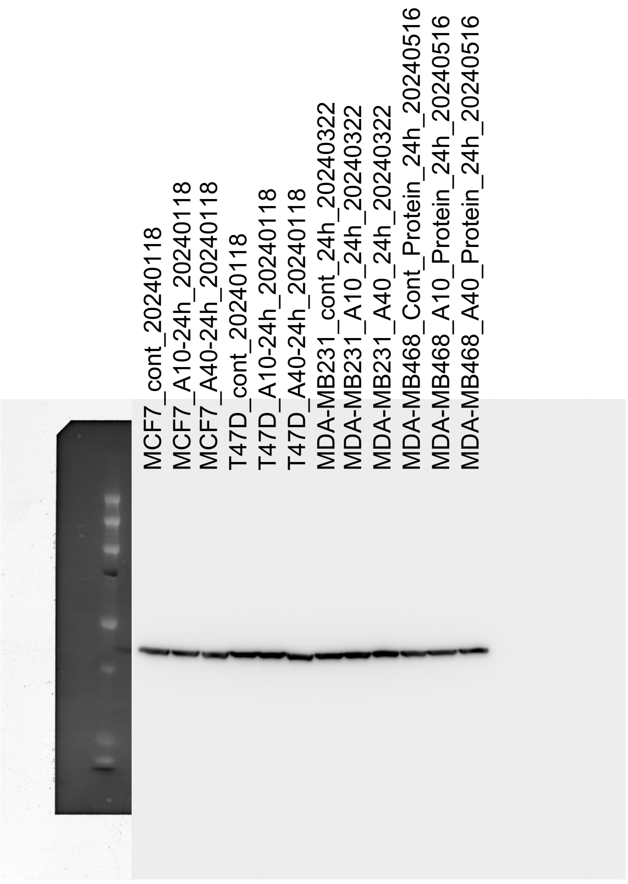

Figure 4C

2024/9/19_P322_No.141_HO-1/HMOX1_CST43966

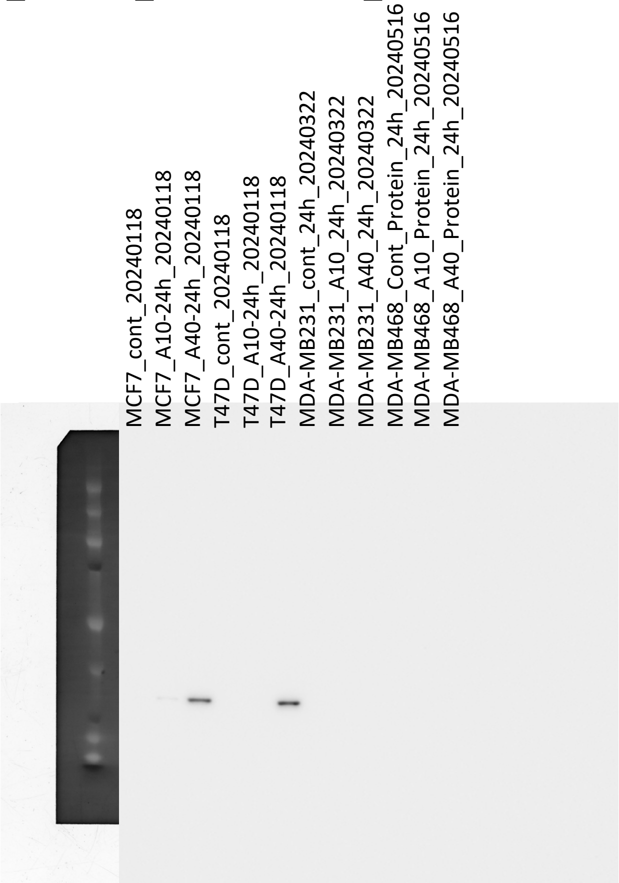

2024/10/31_P322_No.141_bactin

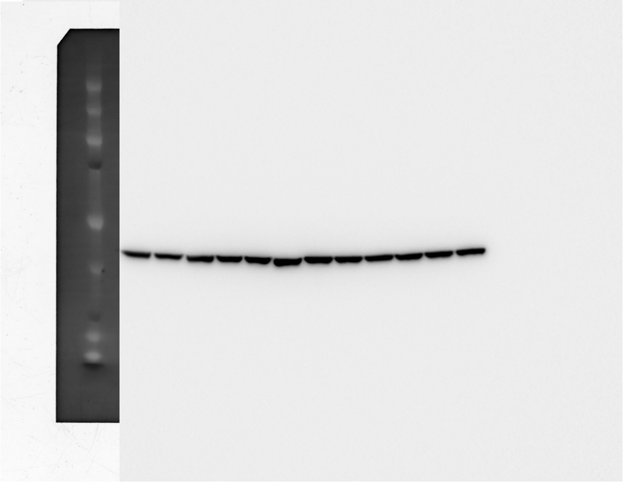

Figure 4C

2024/8/15_P309_No.139_CHOP/DDIT3_CST2895

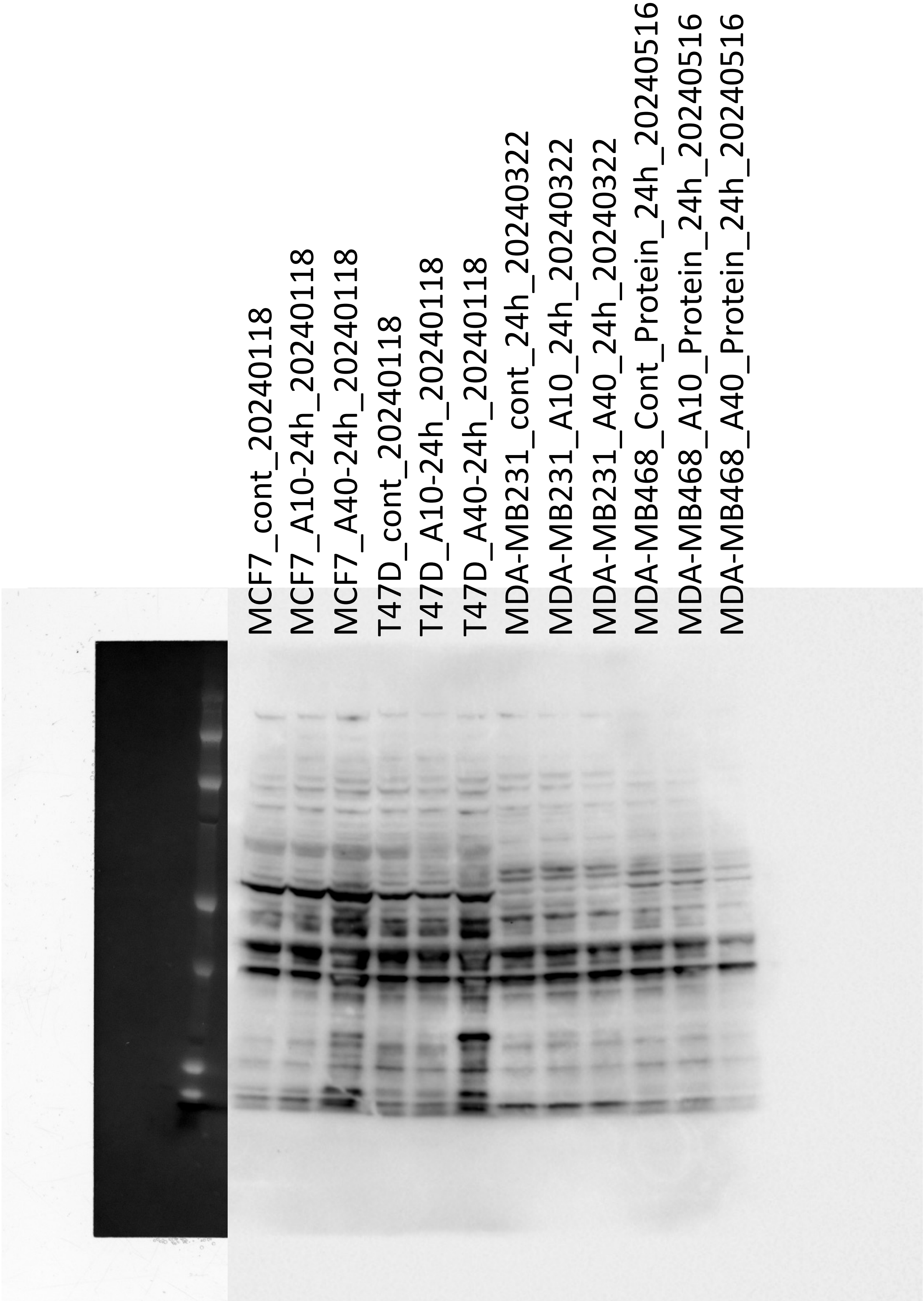

Figure 4C

2024/8/20_P309_No.139_IRE1a

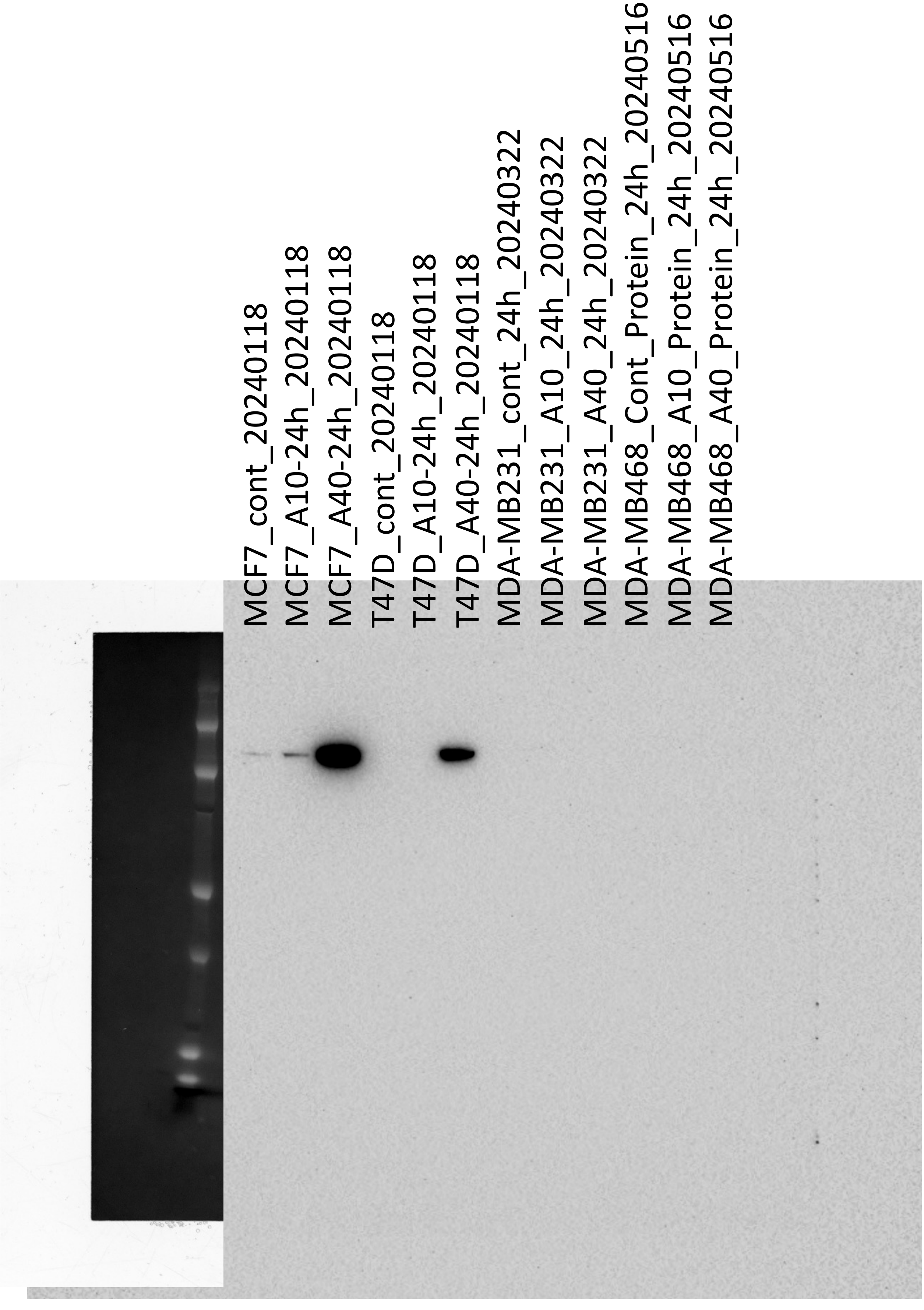

Figure 4C

2024/9/4_P309_No.139_bactin

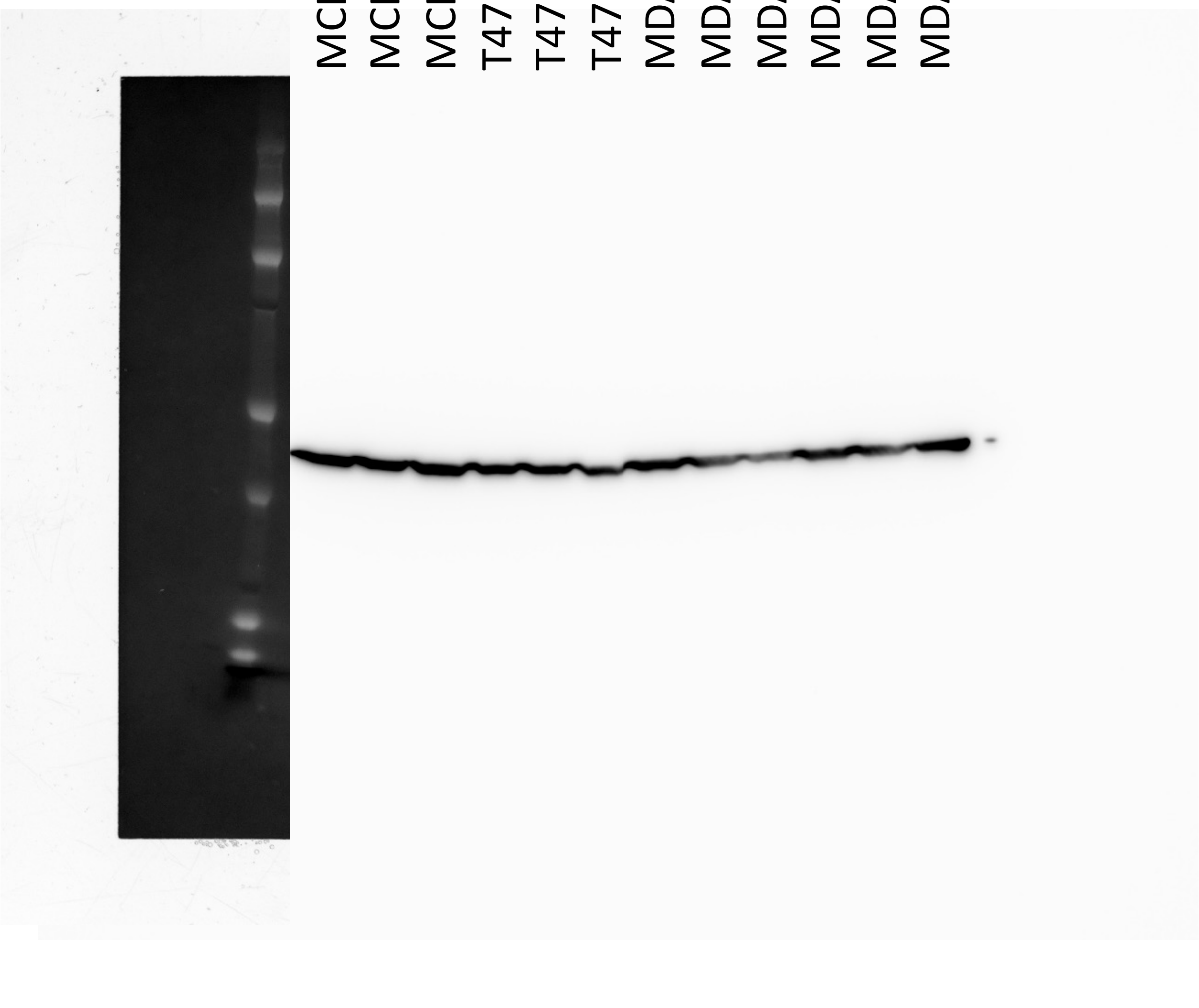

20250109_P373_no158_b-actin_cst4970

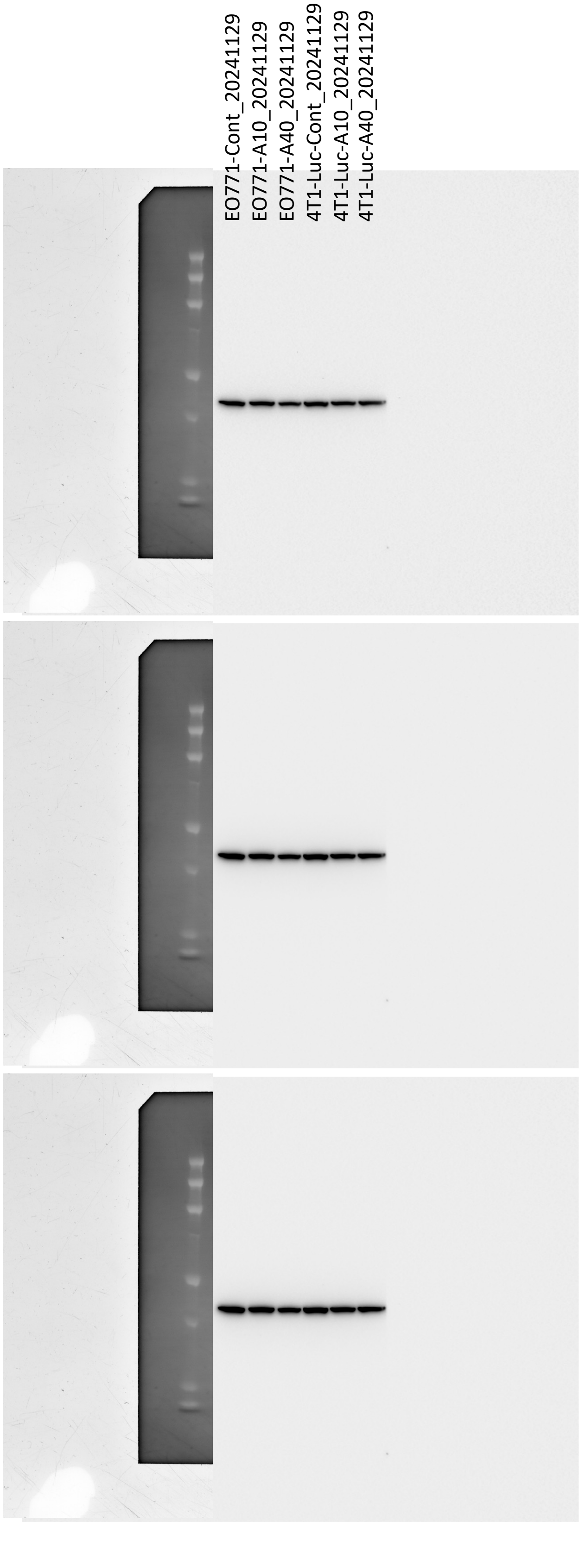

20250109_Cell lines_b-actin_cst4970

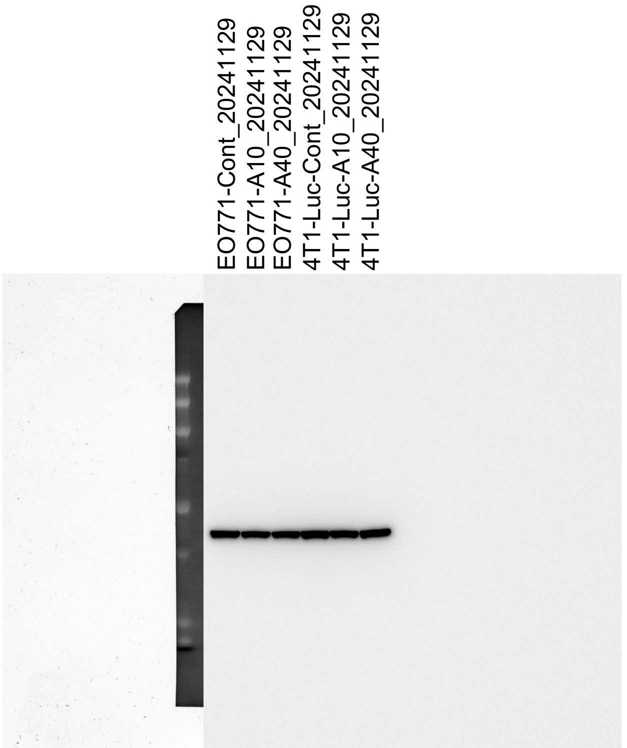

20241211_cell lines_b-actin_cst4970

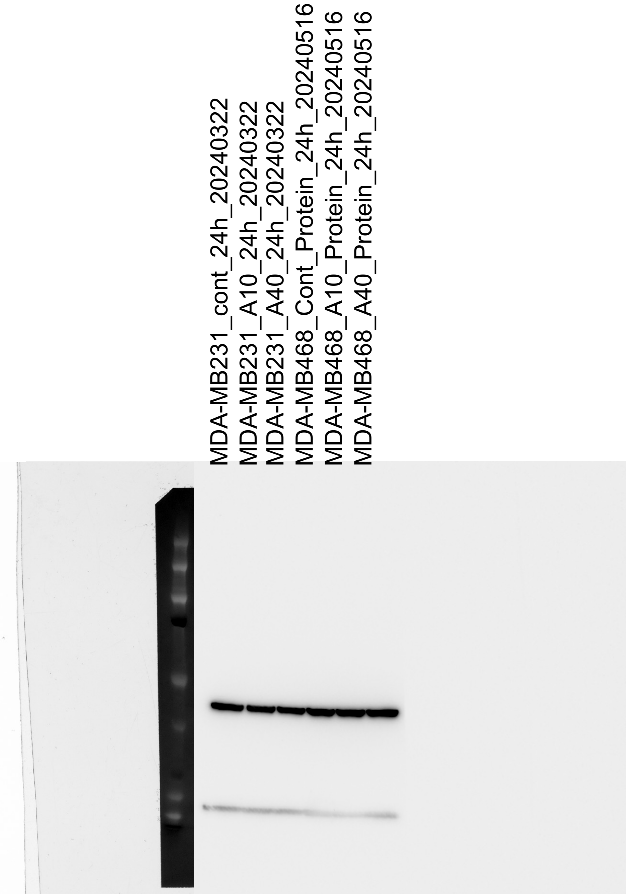

20241211_P342_no150_b-actin_cst4970

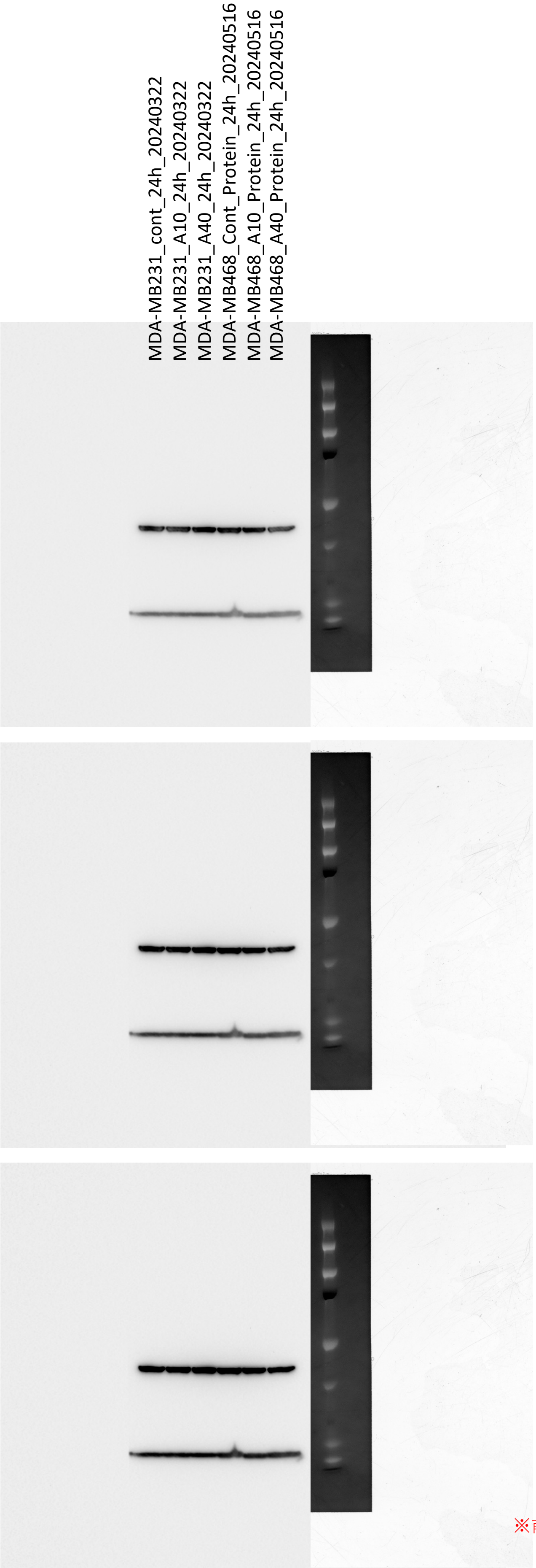

※前回検出のバンドが見えている

20241211_P342_no151_b-actin_cst4970

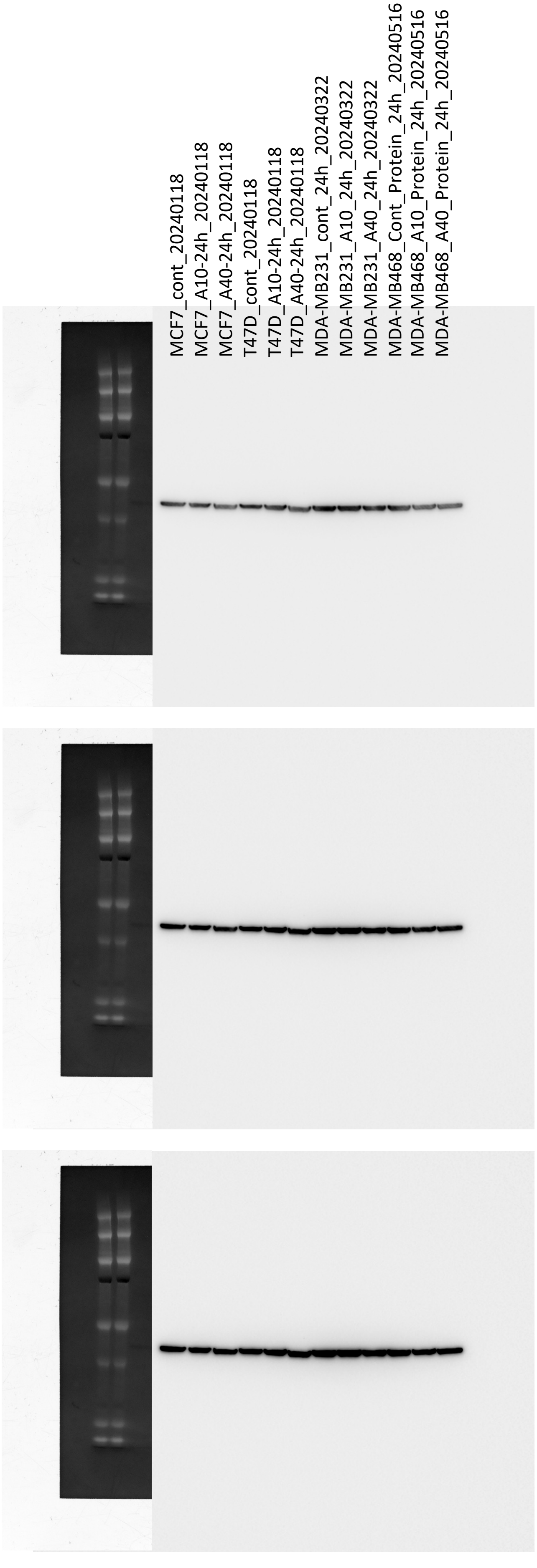

20241112_P330_no143_b-actin_cst4970

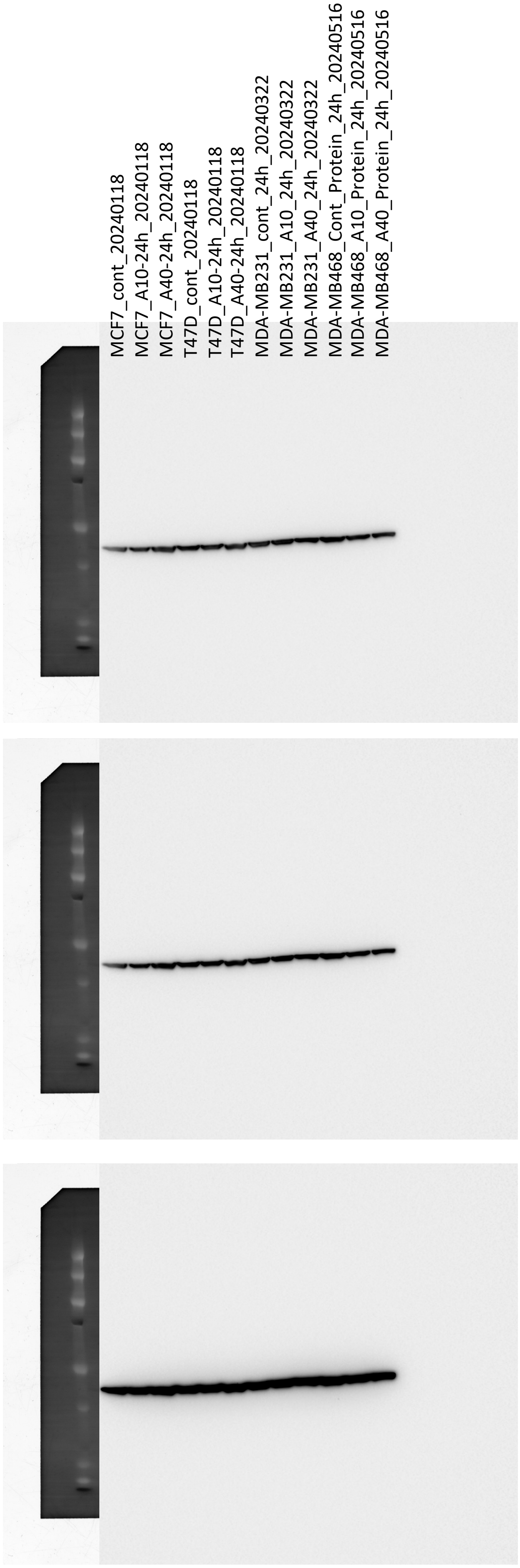

Supplement: Supplementary file 8 — all raw_western_blot_images_of_main_figures [file 41419_2026_8583_MOESM8_ESM.pdf]
