# Supplementary material for: AdipoR1–AMPK axis suppresses breast cancer across molecular subtypes via multimodal cell death pathways, including ferroptosis and apoptosis
Source: Cell Death Dis. 2026 Mar 26;17(1):384. doi: 10.1038/s41419-026-08583-7 (PMC13049035; doi:10.1038/s41419-026-08583-7)

Supplementary Figure S2

A OXIPHOS Ab cocktail:ATP5A and UQCRC2

20251204_P629_No193_OXPHOS_ab110411_Prime

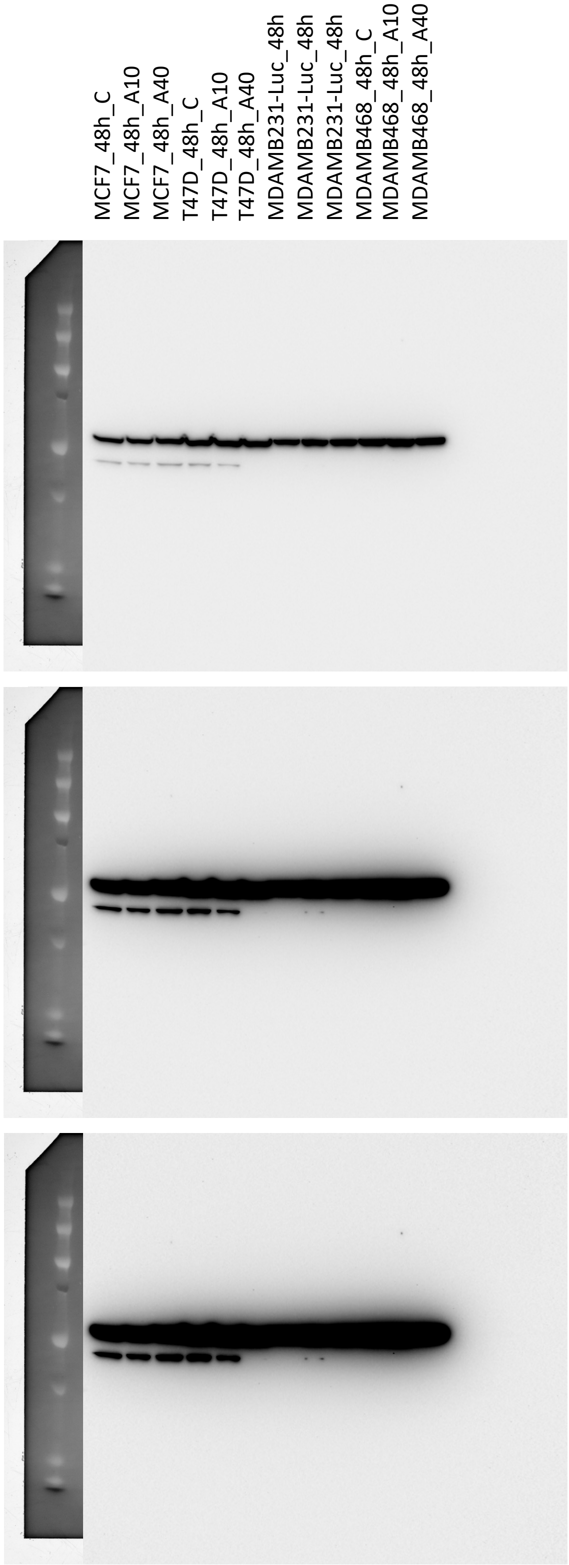

Supplementary Figure S2

A OXIPHOS Ab cocktail: SDHB and MTCO2

20251121_P618_No189_t-OXPHOS_cst110411_prime

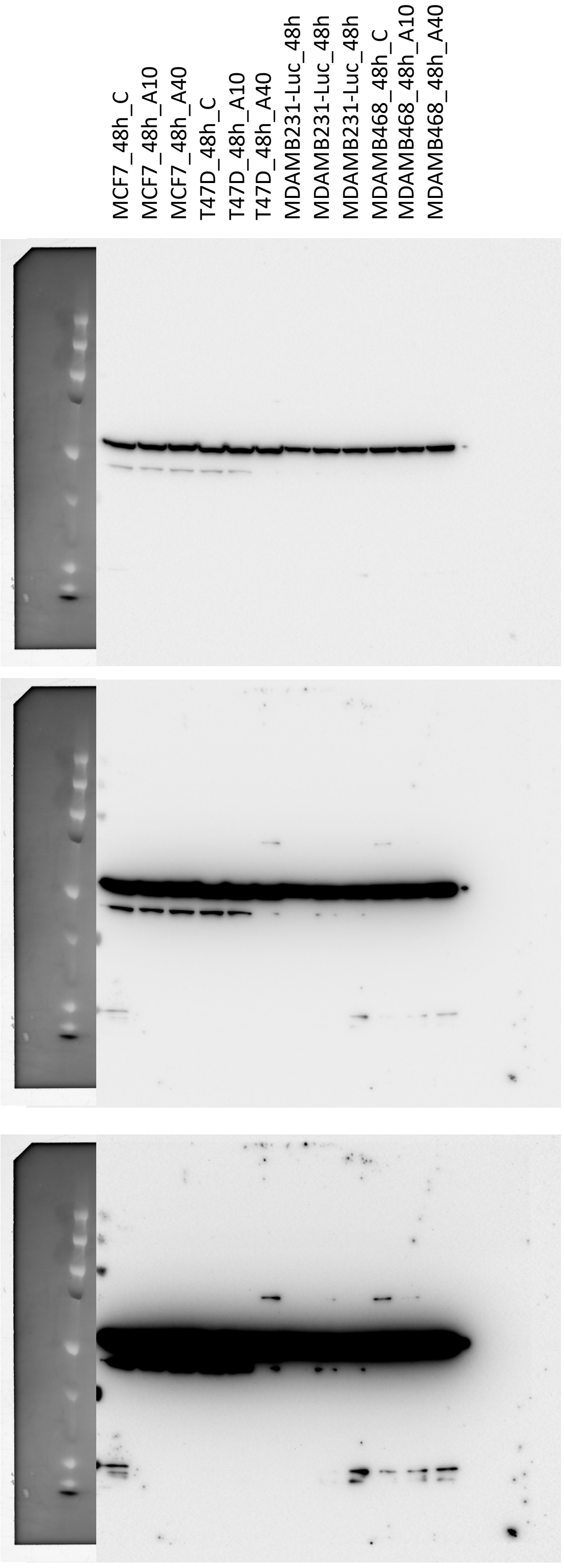

Supplementary Figure S2

A Tom20

20251126_P619_No191_Tom20_cst42406

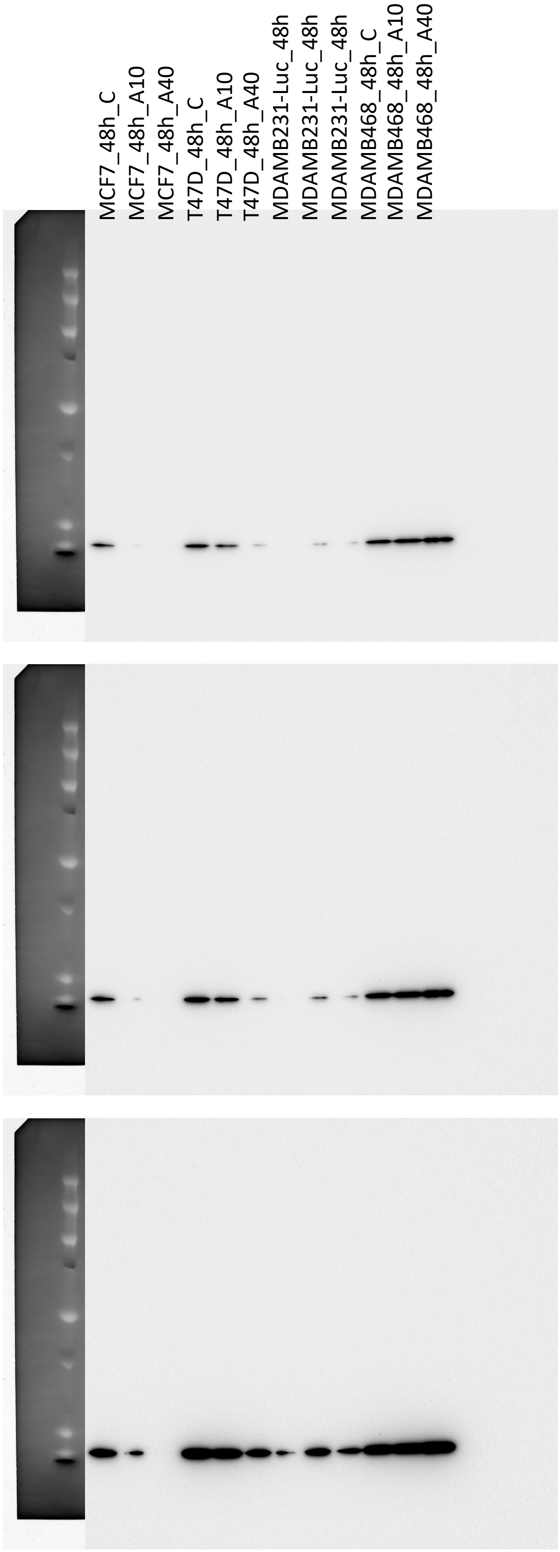

Supplementary Figure S2

A UCP2

20251128_P618_No189_UCP2_CST89326

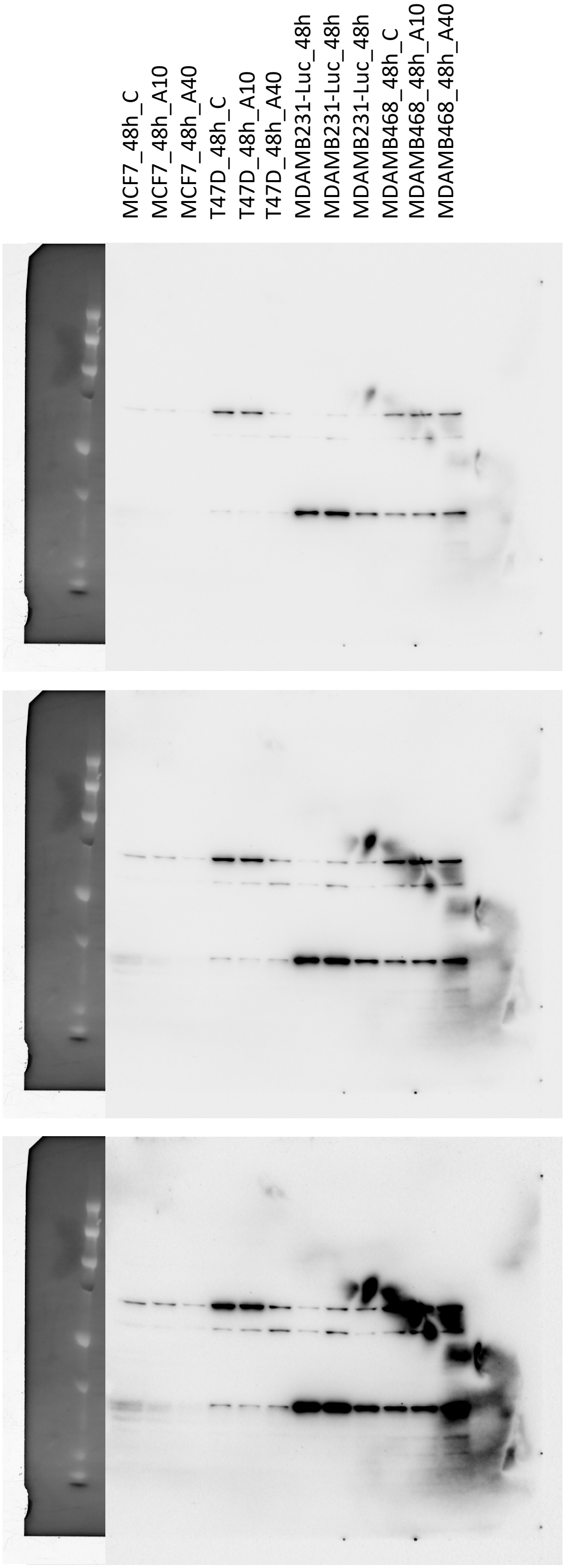

Supplementary Figure S2

A **β-actin**

20251203_P619_No191_b-actin_cst4970

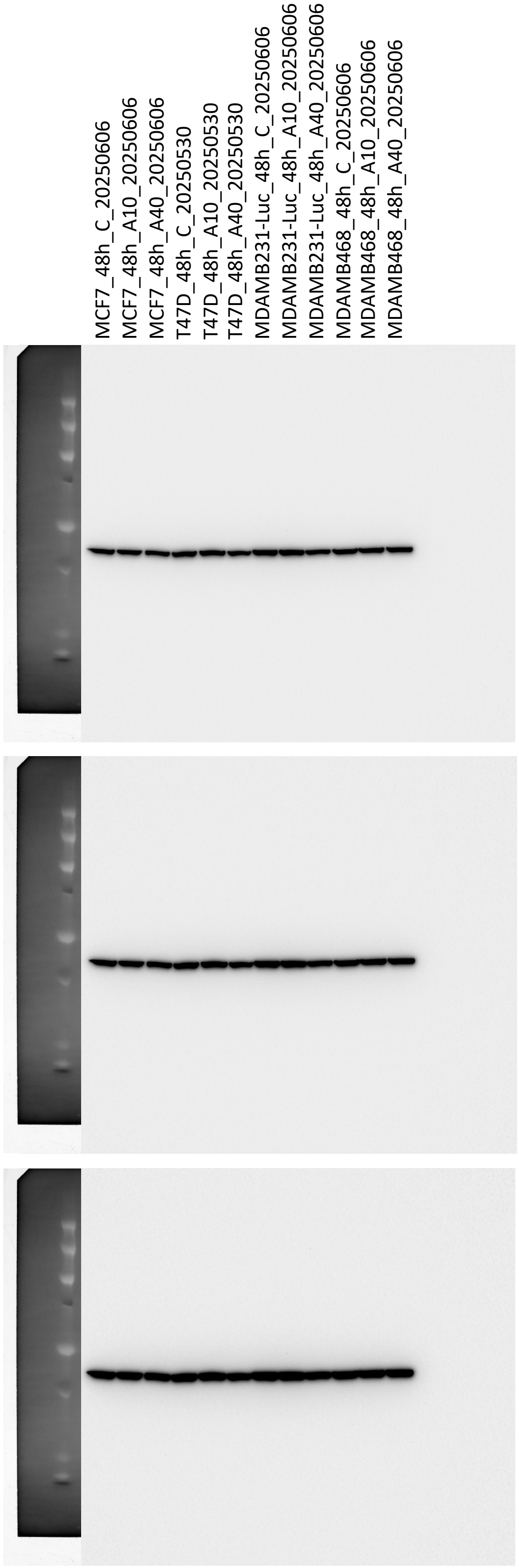

Supplementary Figure S5

C BRCA1

20250710_P487_No181_BRCA1_CST50799

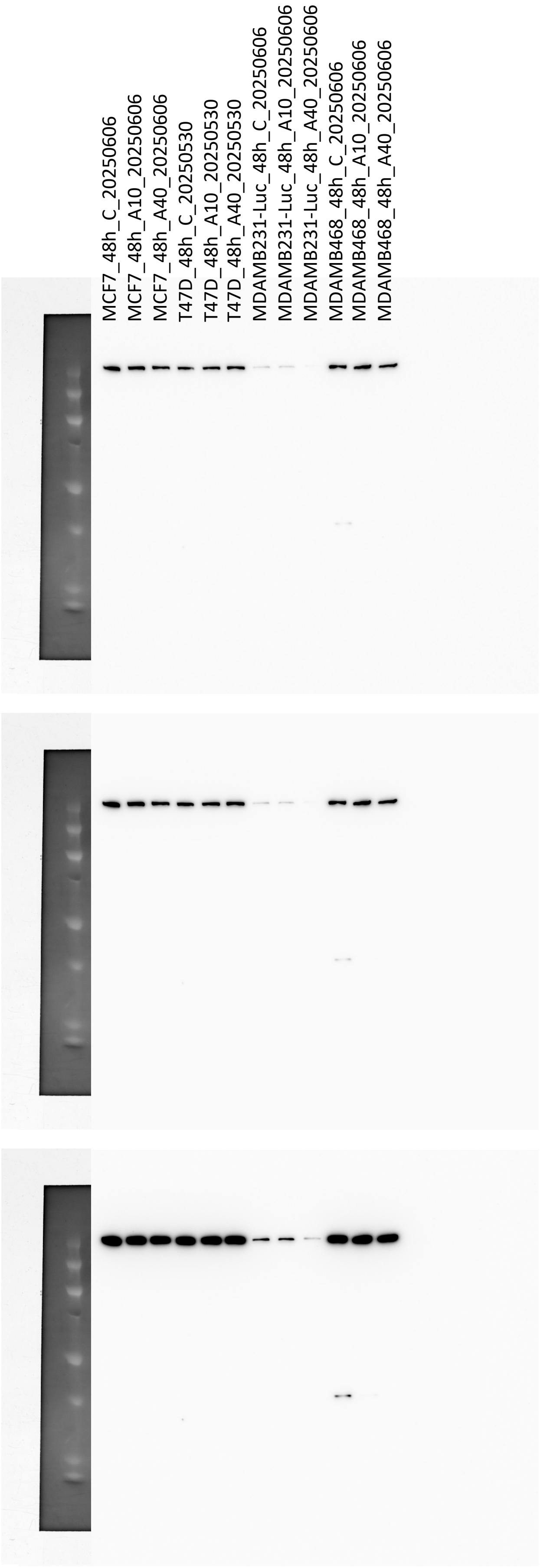

Supplementary Figure S5

C β-actin

20250731_P487_No181_b-actin_cst4970

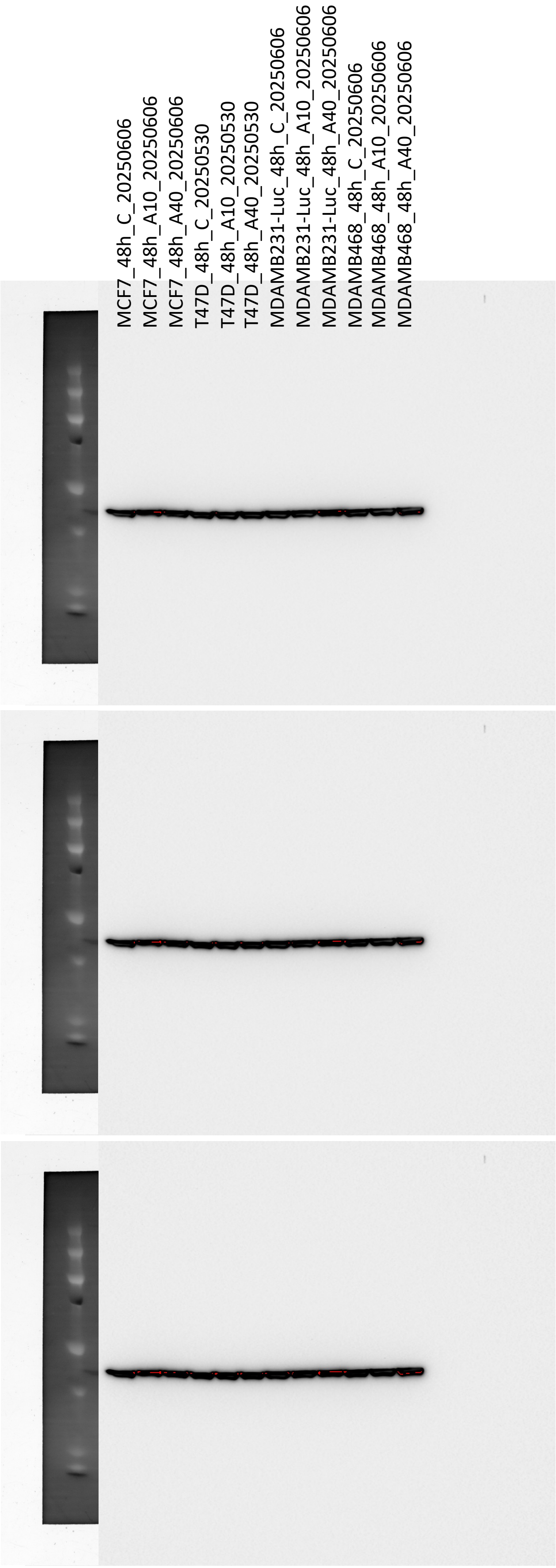

Supplementary Figure S5

D TROP2

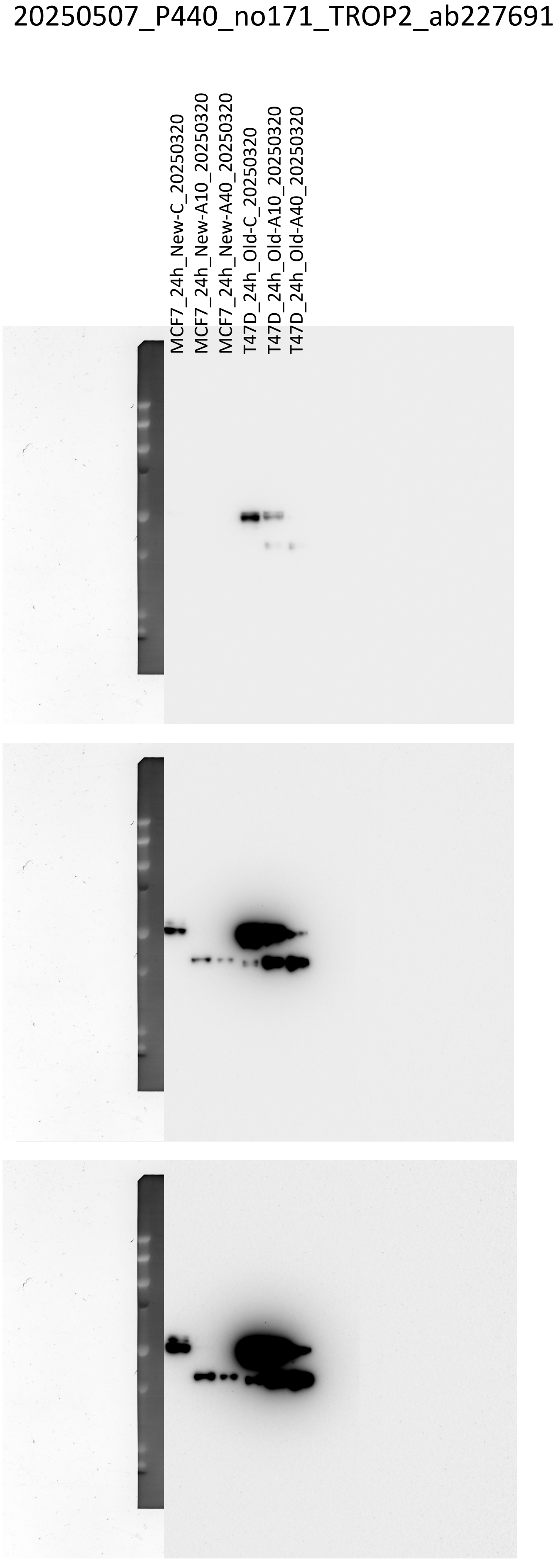

Supplementary Figure S5

D β-actin

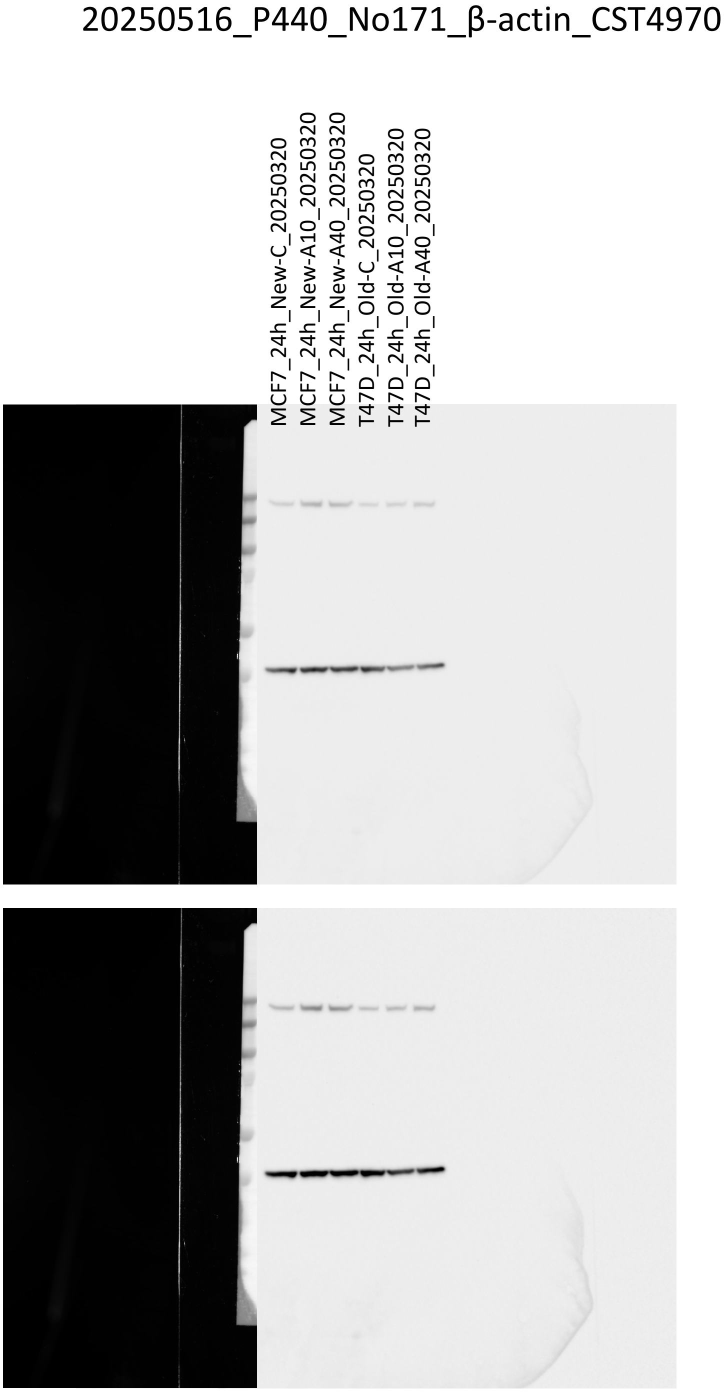

Supplement: Supplementary file 9 — all_raw_western_blot_images_of_supplementary_figures [file 41419_2026_8583_MOESM9_ESM.pdf]
